# Digital Tools to Support Mental Health in Later Life: Scoping Review of Systematic Reviews

**DOI:** 10.1007/s11920-026-01689-x

**Published:** 2026-06-17

**Authors:** Melanie Stowell, Rosie Dobson, Noah Bunkley, Judith McCool, Vili Nosa, Robyn Whittaker

**Affiliations:** 1 School of Population Health, University of Auckland, Building 507, Grafton Campus, 28 Park Avenue, Grafton Auckland, 1023 New Zealand; 2 Planning, Funding and Outcomes, Te Whatu Ora | Health New Zealand, Auckland, New Zealand

**Keywords:** Older adults, Mental health, Psychosocial health, Digital health, Telemedicine, Technology interventions

## Abstract

**Purpose of Review:**

This scoping review synthesises existing evidence from systematic reviews on the effectiveness and implementation of digital mental health interventions among community-dwelling older adults.

**Recent Findings:**

Twenty-one systematic reviews were included. Results showed that a range of digital tools demonstrate potential to improve common mental health and psychosocial symptoms among older adults, with most evidence concentrating on digital tools to improve depressive symptoms. However, reviews’ findings were frequently mixed and accompanied with cautions that primary evidence under-reported key elements such as theoretical underpinnings, intervention design process, participant demographics, intervention acceptability and usability, participant retention, adverse events, and long-term outcomes.

**Summary:**

More rigorous research and reporting are needed to understand the mechanisms underpinning effective digital mental health interventions for older adults and how they might mitigate the age-related digital divide in mental health services.

**Supplementary Information:**

The online version contains supplementary material available at 10.1007/s11920-026-01689-x.

## Introduction

Populations around the world are ageing at an accelerated pace [1]. While older adults aged 65 + years comprised just 5% of the global population in 1950, this proportion is expected to rise to 16% by 2050 [2]. Much of this population is concentrated in the Western Pacific region, which is home to over one-third of the 700 million people aged 65 + globally [3]. The efforts leading to increases in life expectancy are to be celebrated, however many have observed that longer lives are not necessarily healthier [4]. Indeed, more people are spending more years living with complex health and social care needs, which has significant implications for health systems and economies [4]. Health systems are typically better equipped to address acute conditions and urgently need to adapt to be able to address the long-term care needs that are common in a rapidly growing population group [5].

Mental health is an integral component of one’s total state of health and may be both a cause and effect of poor physical health [6]. Later life in particular may be a time of greater social isolation, poorer mental health, and propensity for substance use [7]. Mental health risks may be compounded if an older person experiences multiple intersecting identities (such as belonging to an ethnic minority group, Rainbow community, or living rurally) which lead to cumulative experiences of disadvantage and discrimination [7, 8]. The complex effects of intersectionality on mental health outcomes among older adults is still an emerging and important area of research [9–12]. People of all ages living with mental health conditions experience higher rates of disability and mortality [6] and among older adults mental health conditions account for 11% of total disability [7]. Over one-quarter of global deaths from suicide are among people aged 60 + years [7], highlighting possible unmet needs among this population.

In addition to facing specific risk factors for mental health problems, older adults also experience unique barriers to seeking and receiving support in already-strained mental health and addiction systems. These include low perceived need, fear of stigma, financial cost, and lack of engagement in primary care [13–15]. Alternative solutions to traditional psychiatric services have been endorsed to address these barriers and provide mental health support to older adults before they reach a crisis point [7, 13].

Digital health tools have potential to improve the provision of timely, sustainable and cost-effective services, including brief interventions to support mental health [16, 17]. However, research has highlighted inequitable access to digital services, particularly among older adults [18]. As the availability of health-supporting technologies rapidly evolves, older populations are frequently overlooked in the development of accessible and effective digital services [19]. This results in a ‘digital divide’ and potential worsening of unaddressed conditions [20], particularly in a post-Covid pandemic world in which provision of digital healthcare continues to advance [21]. Researchers, policymakers and service providers must have a comprehensive understanding of the mechanisms needed to engage older adults in digital mental health to ensure this important population group receives equitable access to service provision.

This umbrella review sought to provide an overview of existing evidence on the effectiveness and implementation of digital mental health interventions among community-dwelling older adults.

## Methods

### Design

A scoping ‘review of reviews’ approach was chosen following an initial scoping which revealed numerous systematic reviews covering the topic. Providing an overview of the evidence from a large body of literature helps to minimise duplication and research waste [22]. Scoping review methods originally proposed by Arksey and O’Malley [23] and recently updated [24–26] were used as they permit a rigorous mapping of the evidence and identification of evidence gaps. The review was informed by the PROGRESS-Plus framework to ensure that equity is considered across the spectrum of factors that may impact on older adults’ access to/benefit from digital health tools [27, 28]. Our methods are reported in accordance with the Preferred Reporting Items for Systematic Reviews and Meta-Analyses extension for Scoping Reviews (PRISMA-ScR) checklist [29] and the protocol was not published.

### Search Strategy

A comprehensive search strategy was developed with support from an information specialist, pilot-tested and refined when necessary. Searches were carried out in five databases: APA PsycINFO, Cochrane Database of Systematic Reviews, Embase, Ovid MEDLINE, and Web of Science and incorporated subject headings, synonyms, and text word searching. Reference lists from included reviews were checked for additional eligible articles. See Supplementary File 1 for an example of the search strategy applied to MEDLINE.

### Review Criteria

Inclusion criteria for the review were based on PICOS (population, intervention/exposure, comparator, outcome, study design):

- Population: Older adults (aged 65 +) with mental health or psychosocial risk factors who are community-dwelling.
- Exposure: Studies in which a digital health tool or intervention was developed/adapted and tested with older adults to support their mental/psychosocial health.
- Comparator: Any comparator or no comparator.
- Outcome: Any measure (quantitative evidence) or report of experience (qualitative) of the development, final design, acceptability, and mental health outcomes of the tool’s use with the target population.
- Study design: Qualitative or quantitative systematic reviews (those that meet 4 of 5 Database of Abstracts of Reviews of Effects [DARE] criteria) [30] of primary studies from any country, in English, in the last 17 years (2009–2024). The initial search conducted in 2024 included articles from the last 15 years (January 2009–7 May 2024) and was subsequently updated to include articles published from May 2024–9 April 2026.

Reviews reporting on digital mental health tools for both younger (18–64 years of age) and older adults (defined as age 65 + years by the Organisation for Economic Co-operation and Development [31]) were only included if older adults aged 65 + years were explicitly targeted or if the average age of the study sample was 65 + years. Eligible reviews included digital tools designed to support individuals with mental health or psychosocial risk factors (i.e., loneliness, social isolation), who were community-dwelling. Reviews not reporting participant residence status or that focused on studies about older adults living in care (i.e. hospitals, care homes) were excluded.

Digital tools designed to address physical or neurological conditions were excluded, unless they explicitly targeted mental health concerns related to these. Similarly, if a digital health intervention targeted both physical and mental health conditions, studies were included if the results specific to mental health components were discernible. Dyadic/family interventions were included. Interventions targeting specific behaviours (e.g. medication reminders) or whose primary focus was diagnosis, symptom assessment or patient monitoring were excluded.

Included digital tools were any electronic, mobile, sensor-based or online-delivered health intervention [32]. Reviews assessing multimodal interventions were included if findings for the digital aspects were discernible in the results.

### Study Selection

Titles and abstracts were screened within Rayyan [33]. Full texts of selected records were retrieved and assessed against the inclusion criteria. Results were managed in Endnote [34]. Ten percent of records were double-screened at each stage (MS and NB) and any disagreements at each stage resolved through group discussion and consensus [35]. Remaining records were screened by a single reviewer (MS).

### Data Extraction and Synthesis

A data extraction form was developed and piloted. Information from primary studies meeting the review eligibility criteria was extracted and important overall learnings were also considered where relevant. Key study information for each source of evidence were extracted: review author, year published, countries included, study population, mental health concerns being addressed, methods and follow-up timeframes, type of intervention, and main relevant outcomes of the intervention. Main findings of each review synthesis were summarised, including findings related to population subgroups.

Additionally, a narrative synthesis about the digital tool development process, evaluation, and implementation was undertaken to examine the extent to which interventions were developed to meet the needs and preferences of older adults. Measures of interest included reviews’ reporting of primary study quality/risk of bias, theoretical underpinnings, participatory design, attrition/retention rates, usability and acceptability outcomes.

### Quality Assessment

Reviews were appraised using a modified Critical Appraisal Skills Programme (CASP) checklist for systematic reviews [36]. The degree of overlap of single studies across included systematic reviews was also assessed to consider the risk of overweighting findings [37].

## Results

**Fig. 1:**
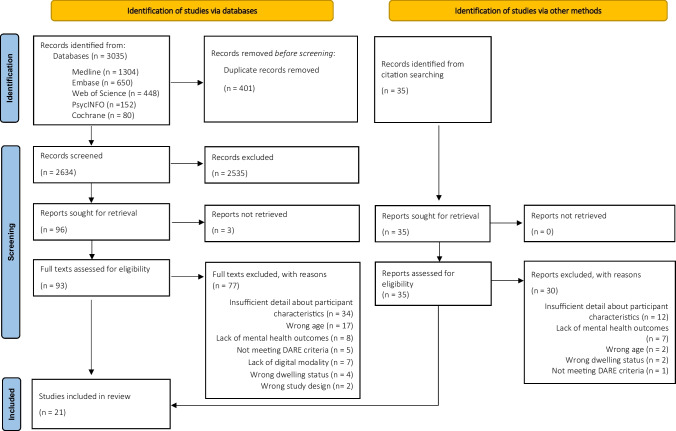
Preferred Reporting Items for Systematic Reviews and Meta-Analyses (PRISMA) flow chart

Twenty-one articles met the criteria for inclusion [38–58] of which 16 were identified from databases searches and five through reference lists (Fig. 1). Table 1 provides a summary of the reviews.

**Table 1:** Characteristics of included systematic reviews (*N* = 19)^a^

Author	Year	Review type	Review objective	Countries included	Target population	Types of interventions included	Intervention methods included	Symptoms being assessed	Theoretical underpinnings/approaches	Study sample sizes and durations	Key mental health findings
Chae & Lee	2022	Systematic review and meta-analysis	To examine the effectiveness of information and communication technology‐based cognitive interventions in community‐dwelling older adults with mild cognitive impairment or mild dementia	Hong Kong Italy Portugal	Community‐dwelling older adults with mild cognitive impairment or mild dementia	ICT cognitive training interventions	RCT	Depression	Cognitive training	11–51 4–6 weeks	GDS scores showed a statistically significant reduction in depression with the ICT‐based intervention
Chastin et al	2021	Systematic review and meta-analysis	To evaluate the effectiveness of interventions aimed at reducing sedentary behaviour amongst older adults living independently in the community compared to control conditions	USA	Community-dwelling older adults with ability to exercise but reporting < 60 min per week of moderate-to-vigorous physical activity	Multimodal intervention consisting of in-person and phone consultations + use of digital armband and personal smartphone interface	RCT	Depression	The 1 eligible primary study did not report using a behaviour change theory	38 12 weeks	No difference between groups in depressive symptoms at follow-up
Christensen et al	2020	Systematic review and thematic analysis	To conduct a systematic review of the existing research literature, focusing on patients’ and providers’ experiences of video consultations used in the treatment of patients 60 + years with unipolar depression	Canada USA	Older adults with unipolar depression	Video and tele-therapy and counseling	Interventions assessing feasibility and efficacy	Depression	Not reported by review	4–42 4–12 weeks (duration not reported in 1 study)	Mixed but moderate to high acceptance, positive attitudes and satisfaction with teletherapy formats. Mixed/conflicting results regarding teletherapy’s implications for privacy
Cremers et al	2019	Systematic review and qualitative synthesis	To investigate the effectiveness of a broad range of low-intensity, psychological interventions on the mental health of older adults through systematic evaluation of the extant literature and to suggest clinical implications for the findings	Australia Canada Sweden UK	Older adults with mild to moderate mental health problems	Internet-based CBT	RCT; controlled trial; pre-post feasibility trial	Anxiety; depression	CBT	22–33 7–8 weeks	Low-intensity psychological interventions can be effective for older adults with mild-to-moderate mental health problems (significant improvements were found), but generalizability is constrained
de Oliveira et al	2022	Systematic review and meta-analysis	To evaluate the efficacy of interventions by telemedicine on reducing depressive and anxious symptoms or disorders in older people through a systematic review and meta‐analysis	Australia Canada China Israel Italy Sweden USA	Older adults without dementia	Telemedicine interventions addressing depressive and/or anxious symptoms	RCT; quasi-experimental study	Anxiety; depression	CBT, behavioural activation	21–1018 3.5–48 weeks	Telemedicine interventions are feasible; several studies demonstrated significant improvement in depressive or anxiety symptoms
Dworschak et al	2022	Systematic review and meta-analysis	To conduct a systematic review and meta-analysis on the existing literature on internet-based psychological interventions for the treatment of common mental disorder symptoms and psychosocial problems in older adults	Australia Canada Germany Sweden UK USA	Older adults	Internet-delivered and home-based interventions	RCT; pilot RCT; controlled trial	Common mental disorder and psychosocial symptoms	CBT, positive psychology, mindfulness	40–433 2–10 weeks	Significant large effect of internet-based interventions for older adults were found for overall symptom severity (depression, anxiety, PTSD, stress) and for depression symptom severity. No significant effects were found for anxiety symptom severity
Fernandes et al	2025	Systematic review	To synthesize current evidence on telehealth interventions for adults aged 65 years and older, focusing on their effects on health outcomes, quality of life, and well-being	Brazil Cyprus Germany Italy Japan	Older adults aged 65 +	Telehealth interventions	RCT; quasi-experimental study; prospective cohort study	Depression; anxiety; stress; resilience; loneliness	Not reported by review	29–96 8–52 weeks	Improvements in all mental health areas but effect sizes/significance not reported
Goodarzi et al	2023	Systematic review and qualitative synthesis	To conduct a systematic review of RCTs reporting the efficacy of virtual interventions for reducing symptoms of depression in older adults	Australia Canada Israel Sweden USA	Community-dwelling older adults with multiple comorbidities	Virtual (internet-based, video or telephone) self-directed or supported CBT	RCT	Depression	CBT	40–1018 8–94 weeks	Included studies demonstrated feasibility of interventions such as internet or telephone CBT with some papers showing statistically significant improvement in depressive symptoms
Jin et al	2021	Systematic review and meta-analysis	To systematically analyze the effectiveness of technology-based interventions for reducing loneliness in older adults	Israel UK	Older adults	Technology-based interventions where a core component involved use of technology to reduce loneliness	RCT	Loneliness	Not reported by review	39–95 12–15 weeks	Interventions had little or no effect on loneliness reduction in older adults; subgroup analyses (computer/internet-based vs smartphone video conferencing) also had very uncertain results
Li et al	2025	Systematic review and meta-analysis	To evaluate the effectiveness and implementation of digital health interventions compared with control group for older adults with cognitive frailty	Taiwan China	Older adults with cognitive frailty (coexistence of physical frailty and cognitive impairment)	Video and digitally based physical exercise interventions	RCT; non-randomised study of interventions	Loneliness; depression; social connectedness; social isolation	Biomechanical Frame of Reference, the Compensatory Frame of Reference, the Person-Environment-Occupation Model, and Intergenerational Program	69–202 8–18 weeks	Interventions had a significant effectiveness on improving depressive symptoms; Meta-analysis for loneliness was not performed due to insufficient intervention data
Morris et al	2014	Systematic review and critical evaluation	To examine the effectiveness of smart technologies in improving or maintaining the social connectedness of older people living at home	Netherlands Norway USA	People aged 45 + years living in their homes	‘Smart technology’ interventions including web-based information, intervention and communication programs	RCT; Cohort study	Social connectedness; depression	Not reported by review	14–100 10 weeks −36 months	Mixed but mostly significant results among the large number of outcome measures including loneliness, social contacts, depression
Oh et al	2026	Systematic review	To investigate the impact of AI interventions on loneliness and social isolation in older adults	Netherlands USA	Older adults with loneliness or cognitive impairment	Conversational AI, chatbots, or conversational agents	RCT; pilot feasibility study	Loneliness; depression; anxiety	Not reported by review	10–32 8 weeks (when reported)	No decrease in loneliness (χ22 = 0.02, *p* =.99) Depression decreased – however anxiety increased (potentially due to thought of losing digital companion)
Posadzki et al	2016	Systematic review, narrative synthesis, and meta-analysis	To assess the effectiveness of automated telephone communication systems for preventing disease and managing long-term conditions on behavioural change, clinical, process, cognitive, patient-centred and adverse outcomes	USA	Older adults recruited from a longitudinal study	Telephone-Linked Computer-based Long-term Interactive Fitness Trainer	RCT	Depressive symptoms	Social cognitive theory	103 12 months	The intervention may have reduced depressive symptoms (*P* = 0.030)
Poscia et al	2017	Systematic review and qualitative synthesis	To summarize and update the current knowledge on the effectiveness of the existing interventions for alleviating loneliness and social isolation among older persons	Netherlands UK	Older adults	Video/voice network, telephone befriending, internet use	Pre-post study; parallel group RCT	Loneliness; social isolation	Not used in the relevant primary studies	58–440 6 weeks-12 months	Mixed but mostly significant results among outcome measures including loneliness, social contacts, mental wellbeing
Pu et al	2017	Systematic review and meta-analysis	To summarize the effectiveness of social robots on outcomes (psychological, physiological, quality of life, or medications) of older adults from RCTs	Japan New Zealand	Older adults	Social robot interventions	RCT	Depressive symptoms	Not reported by review	29–40 6–8 weeks	No statistically significant effects for depressive symptoms
Rai et al	2022	Systematic review and deductive thematic analysis	To collate and summarize current evidence for digital technologies to prevent social isolation and loneliness for people with dementia	Spain USA	People with mild cognitive impairment/dementia	Digital companion, robot, TV-delivered support	Intervention studies of unspecified design	Social isolation and loneliness	Participatory arts model	10–93 12 weeks (duration not reported in 1 study)	Findings showed improvements in mood/depression, loneliness, possible reductions in isolation (significance not reported) 1 study found increased levels of anxiety in participants with high reported levels of attachment to the digital companion Purchase availability was assessed and found for 1 relevant primary study (USD $9,000)
Riadi et al	2022	Systematic review and narrative synthesis	To gain insight into the designs and aspects of digital mental health interventions for older adults, and to synthesise methodological findings from RCTs with older adult participants to extract important information to conduct future RCTs for this population	Australia Germany USA	Older adults with depressive or anxious symptoms	Virtual reality hardware, online course, internet-delivered CBT, virtual physical activity	RCT	Depression; anxiety	CBT	20–51 6 weeks-6 months	Mixed results with none or some improvements in depressive and anxiety symptoms (non-significant or significance not reported for eligible studies)
Ronzi et al	2018	Systematic review and narrative synthesis	To synthesise the evidence of health impacts of interventions on respect and social inclusion in older people	Netherlands USA	Older adults living in the community	Computer/information and communication technology training programmes	RCT; controlled before-and-after study	Anxiety; Depression; sleep complaints	Not reported by review	7–191 Durations unclear for relevant studies	Non-significant reductions in depression and anxiety; non-significant improvement in mental health overall. Perceived positive impacts on social wellbeing from 1 qualitative study
Shah et al	2021	Systematic review and meta-analysis	To assess the effectiveness of digital technology interventions in reducing loneliness in adults	Netherlands Sweden USA	Adults aged 18 + years	Digital technology interventions including TV-delivered support, social activities via social websites, PRISM system (personal reminder information and social management)	RCT; randomised crossover trial; controlled before-and-after study	Loneliness	Not reported by review	30–300 3–12 months	Narrative summary showed a reduction in loneliness for intervention groups, but meta-analysis showed insignificant effects. Confidence intervals and quality of evidence leave room for uncertainty about effects
Silva et al	2018	Systematic review and meta-analysis	To assess the effectiveness of technology-mediated dance interventions and their impact on psychosocial factors in older adults	Australia Switzerland	Older adults aged 65 + years, with or without chronic conditions	Dance interventions mediated by a form of technology	RCT; quasi-RCT with a compared with a control group	Depressive symptoms	Not reported by review	32–90 2–6 months	No significant improvements in symptoms of depression
van den Berg et al	2012	Systematic review and narrative synthesis	To analyze whether telemedical healthcare concepts are suitable as a new innovative option in medical care for older patients	USA	Older patients with a defined diagnosis or medical need	Telemedicine between a medical provider and patient in their own home	RCT	Depression	Not reported by review	115 12 months	Intervention group saw improvement in depression symptoms and social functioning (significance not reported)

RCT indicates randomized controlled trial; *CBT* cognitive behavioural therapy; *ICT* information and communication technology; *GDS* Geriatric Depression Scale; *PTSD* posttraumatic stress disorder

^a^ Information from primary studies, where applicable, only extracted if meeting eligibility criteria for the current review

### Review Characteristics

Twelve (57%) included systematic reviews were published in the last five years with the highest concentrations in 2021 and 2022 [38, 39, 41–46, 51, 52, 54, 57, 58]. Just over half of reviews (*n* = 11) conducted a meta-analysis [38, 39, 42, 43, 45, 46, 48, 50, 54, 55] and over one-third (*n* = 8) reported on primary evidence from randomised controlled trials (RCTs) only [38, 39, 44, 45, 48, 50, 52, 56] while the remaining included multiple study designs. Most reviews aimed to assess intervention effectiveness; over one-third also reported a measure of acceptability or feasibility (e.g. satisfaction, usability, retention, adherence, purchase availability; *n* = 8) [40, 41, 43, 46, 51, 55, 56, 58]. Change in mental health symptoms was a primary outcome in most reviews, though in three reviews was a secondary outcome [38, 39, 46]. Most reviews analysed primary evidence about digital health interventions only, while four included mixed modalities [39, 41, 49, 53].

### Intervention Characteristics

Evidence from relevant primary studies most frequently originated from high-income, western contexts including the United States (13 reviews) [39, 40, 42–44, 47, 48, 51–54, 56, 58], Australia (six reviews) [41–44, 52, 55], Sweden (five reviews) [41–44, 54], and Canada (five reviews) [40–44]. Sample sizes from relevant primary studies ranged from four [40] to 1018 [42, 44] participants. Study durations reported by reviews ranged from two weeks [43] to three years [47]. Over half of reviews (*n* = 10) included primary studies targeting older adults with no specific morbidities or symptoms [42, 43, 45, 47–50, 53–55, 57] while the remaining sought evidence from clinical populations or older adults having specific traits such as physical inactivity, cognitive frailty or mild-to-moderate mental health symptoms. Mental health symptoms being assessed were most commonly depressive (*n* = 17) [38–44, 46–48, 50–53, 55–57], anxiety (*n* = 6) [41–43, 52, 53, 57] and psychosocial factors such as stress, loneliness and social isolation (*n* = 10) [43, 45–47, 49, 51, 53, 54, 57, 58]. Interventions varied with most reviews including a range of health interventions classified as digital or information and communication technology-based; several had a narrower focus such as online therapy [41], conversational agents [58] or social robot interventions [50]. Tailoring or personalisation among primary study interventions was documented by six reviews [39, 42, 43, 46, 51, 58] and though not reported in detail included elements such as regular revisiting of individual goals [39] and professional oversight of therapy programming [42]. Theoretical approaches of the primary studies were reported by just over one-third of reviews [41–44, 46, 48, 51, 52], most frequently cognitive behavioural therapy, with some reviews noting that theoretical underpinnings were under-reported by primary studies [48, 51].

### Intervention Findings

Table 2 presents a matrix of the concentration of evidence by type of outcome and mental health symptom. Most reviews reported mixed but overall positive results of primary studies for mental health outcomes, with significant improvements in symptoms reported in 10 reviews [38, 41–44, 46, 47, 49, 57, 58] and mixed results for measures of usability, participant satisfaction/acceptance or retention/engagement when reported [40, 41, 43, 46, 55, 56, 58]. Six reviews reported non-significant intervention effects among eligible primary studies [39, 45, 50, 53–55, 58] while the effect sizes were unclear in the remaining reviews. Two reviews reported on the same primary study which found increased levels of anxiety in a participant with high reported levels of attachment to a digital companion [51, 58]. Other reviews reported issues such as technology frustration and digital literacy challenges [46, 56] and participant attrition possibly reflecting barriers to engagement [55]. One review reported that participants’ perceived usefulness and enjoyment of a conversational AI agent did not predict use [58].

**Table 2:** Review outcomes by mental health symptom^a,c,d^

Type of outcome	Mental health symptom		
	Depression	Anxiety	Psychosocial risk factors^b^
Effectiveness	**▪ Chae & Lee** **▪** Chastin et al ***▪ Cremers *et al** **▪ *de Oliveira *et al** **▪ *Dworschak *et al** **▪ *Fernandes *et al** **▪ *Goodarzi *et al** **▪ Li et al** **▪** Morris et al **▪ Oh et al** **▪** Posadzki et al **▪** Pu et al **▪** Rai et al **▪** *Riadi *et al **▪** Ronzi et al **▪** Silva et al **▪** *van den Berg *et al	**▪ *Cremers *et al** **▪ *de Oliveira *et al** **▪** *Dworschak *et al **▪ *Fernandes *et al** **▪** Oh et al **▪** *Riadi *et al **▪** Ronzi et al	**▪ *Dworschak *et al** **▪ *Fernandes *et al** **▪** Jin et al **▪** Li et al **▪ *Morris *et al** **▪** Oh et al **▪ Poscia et al** **▪** *Rai *et al **▪** Ronzi et al **▪** Shah et al
Satisfaction/acceptance	**▪ Christensen et al** **▪** *Cremers *et al **▪ *Dworschak *et al** **▪** Oh et al **▪** *van den Berg *et al	**▪ Christensen et al** **▪** *Cremers *et al **▪ *Dworschak *et al** **▪** Oh et al	**▪** Oh et al
Retention/engagement	**▪ *Cremers *et al** **▪** *de Oliveira *et al *Dworschak *et al **▪ Li et al** **▪** Silva et al	**▪ *Cremers *et al** **▪** *de Oliveira *et al *Dworschak *et al	**▪** Li et al **▪** Shah et al
Usability	**▪** *Dworschak *et al **▪ Oh et al**	**▪** *Dworschak *et al **▪ Oh et al**	**▪ Oh et al**

^a^ Information from primary sources only displayed if it met eligibility criteria for the current review

^b^ Defined by reviews as stress, loneliness, social isolation, or social connectedness

^c^ Reviews in **bold** reported significant positive results/impacts of the interventions under review

^d^ Reviews in *italics* received a low quality assessment rating (see Supplementary file 2) or included primary studies cited 4 times across reviews (see Supplementary file 3)

### Quality Assessment

Despite most studies receiving an appraisal of good quality (83%; see Supplementary file 2), nearly all (95%) noted quality issues within primary studies due to heterogeneity of study designs, small and homogeneous samples, high attrition, or paucity of relevant primary studies. A check for overlap found that of the 74 relevant primary studies included across reviews, nearly one-third were cited two or more times: three (4%) appeared four times, two (3%) appeared three times, and 17 (23%) appeared twice (see Supplementary file 3). These findings were considered in our interpretation of results.

## Discussion

This research explored the current systematic review evidence about digital tools to support the mental health of community-dwelling older adults. This approach permits a high-level overview of existing evidence for a broad range of digital tools and mental health conditions while also revealing patterns or gaps in the evidence that single reviews and primary studies may miss.

While half the included reviews reported evidence supporting the effectiveness of digital mental health tools in older populations, results were often mixed and nearly always came with caveats about the quality and reporting within primary studies. Primary studies’ under-reporting of important considerations such as theoretical underpinnings, sub-group analyses, long-term outcomes, adverse events and dropout/retention rates was observed in nearly all reviews. Evidence suggests that older adults experience unique barriers to acceptance of health management technology, including functional challenges (e.g. small screens, short battery life), low levels of confidence to engage with devices independently, financial constraints, and privacy/scam concerns [59, 60]. Understanding the generalisability and capacity of digital mental health tools to be safe and effective for the sub-populations in greatest need is crucial to reducing the growing digital divide [61, 62] and thus more rigorous reporting of these components is critical in both primary and secondary research.

Participatory methods such as co-design and personalisation were also noted as missing among primary studies or infrequently discussed in the included reviews, a shortcoming which has been found elsewhere [63, 64]. While it is unclear to what degree end-user involvement in research impacts intervention adoption and effectiveness, some evidence suggests that even low-level end-user involvement provides important learnings for researchers about older adults’ needs, everyday practices, and dispelling age-related stereotypes [65]. This learning has led to study design adjustments and increased sense of participation and autonomy among end-users [65]. Indeed, established guidance for the development of mHealth [66] and other public health interventions [67] emphasises the importance of end-user involvement at every stage to ensure an intervention is useful and accessible to the target population.

Other shortcomings of the evidence included primary studies’ infrequent reporting of cultural considerations and possibility for real-world implementation beyond research settings. Cultural considerations may be explained by the common finding that primary studies were often conducted in high-income countries and, when described, included mostly smaller, educated, abled, ethnic majority participants. This bias has been echoed in research assessing the implementation of digital technologies for older adults across health domains [62]. Notably, one of the included reviews assessed purchase availability of the interventions being tested and found that the real-world cost of a social robot was USD $9,000 at the time of publication [51]. This is important given the evidence that older adults perceive financial cost as a barrier to acceptance of health management technologies [59]. Practical limitations to real-world implementation of digital innovations must be considered as the risks for exacerbating existing inequities is high [64].

An exacerbation of anxiety symptoms was reported in two reviews [51, 58]. This is an important issue to highlight in digital interventions aiming to alleviate mental health symptoms and one that may be undermeasured or under-reported as per findings of the current and other reviews [46, 68]. Others have also discussed the need for more long-term data on the use of technology-based interventions in understanding any unintended negative impacts for older populations [46, 69]. ‘Technology anxiety’ among older adults must be considered when developing digital mental health tools for this population, especially tools that are novel to the older adults tasked with learning to use them [60, 61, 70, 71]. Robust measurement and reporting of adverse effects such as increased anxiety is critical as the number of AI-powered mental health tools grows [72].

### Implications for Research, Policy and Practice

Taken together, these shortcomings suggest a need to improve reporting, user-centred intervention design and implementation focus among digital interventions designed to support older adults’ mental health. Future research should seek to actively involve older adults from varied backgrounds in the research process, collect comprehensive feedback on intervention preferences, acceptability and usability, and report all findings in sufficient detail in both primary and review studies. Practical applications of the resultant intervention should also be considered and reported to ensure possibility for real-world uptake and benefit [16].

Clinicians and organisations must consider the safety, ethics, and accessibility of digital mental health tools for older clients as universal adoption is not recommended. Digital mental health tools may be inappropriate, unethical or harmful for older people experiencing digital exclusion and thus the introduction of these tools into practice must be carefully considered. Given the evidence reported in this review, tools that have been previously validated may nonetheless be trained on data having a risk of sampling bias and thus relevance to other populations and contexts may require further testing. While the interventions reviewed in this work were implemented within research contexts with appropriate data protection measures in place, digital mental health data may introduce new ethical issues in real-world applications, including misuse of sensitive information by third-party companies with access to client data; comprehensive institutional policies will therefore be critical in ensuring the safe and ethical implementation of digital tools [73].

### Strengths and Limitations

This scoping review of systematic reviews provided a pragmatic overview of a large body of evidence, representing an important step in understanding the effectiveness of current digital mental health tools for older populations as well as critical gaps in the evidence. Our inclusion of a range of mental health concerns, therapeutic approaches and delivery modalities means that our findings may be relevant to a broad audience working in this space. Despite these strengths, our scoping review is subject to all the limitations of this approach. While a quality appraisal and assessment of overlapping primary studies permitted consideration of strength of the current evidence, it was out of the scope of this review to conduct a thorough analysis and critically appraised summary. Our reliance on published systematic reviews means that we may have missed relevant elements of the primary evidence that the reviews did not capture. However, nearly all included reviews noted the lack of key elements within the primary studies they investigated and many primary studies received low quality appraisals and high risks of bias.

While we aimed to keep our inclusion criteria broad to provide a comprehensive overview, our criteria excluded reviews of primary studies focusing on care home residents as the needs and supports available to these populations may differ from those living in the community. Future research may seek to compare results for older adults in residential settings to see how this impacts the uptake and effectiveness of digital tools. Similarly, although reviews about digital tools to support older adults with substance misuse were not necessarily excluded from the current review, our search did not explicitly include substance-related terms. Future research should clarify the role of digital tools in addressing substance misuse among older adults.

Our inclusion of only English-language systematic reviews in peer-reviewed journals may have biased our results to Western contexts and relevant work in real-world trials may have been missed. However, our review of reviews included primary evidence spanning 16 countries and results may be relevant to a range of contexts.

## Conclusions

Mental health and digital equity are vital aspects of healthy ageing. This scoping review of systematic review evidence has shown that a breadth of digital tools has been tested among older adult populations to assess their effectiveness for improving several key indicators of mental and psychosocial health. While digital tools show potential to improve symptoms related to depression, anxiety, and psychosocial risk factors such as loneliness and social isolation, issues within the primary studies were frequently reported and there was minimal information available about key elements such as intervention development and participatory design, participant diversity, intervention acceptability and usability, participant retention and engagement, adverse events, and long-term outcomes. More rigorous research and thorough reporting are needed to understand the mechanisms underpinning the most effective digital interventions and how they might work to improve equitable access to effective digital mental health services for older adults.

## Key References

- Kim H-N, Freddolino PP, Greenhow C. Older adults’ technology anxiety as a barrier to digital inclusion: A scoping review. Educ Gerontol. 2023;49(12):1021–38. 10.1080/03601277.2023.2202080.
  - This article synthesizes themes in the literature on technology resistance among older adults and found that research has been done with associated variables such as technology use, perceptions, and intentions, but more research is needed to examine the predictors of discomfort with technology in order to identify effective approaches to resolving technology-related anxiety and hesitancy among older adults.
- Barbosa Neves B, Waycott J, Maddox A. When technologies are not enough: The challenges of digital interventions to address loneliness in later life. Sociol Res Online. 2023;28(1):150–70. 10.1177/13607804211029298.
  - This article discusses sociotechnical challenges of technology-based interventions to address loneliness in later life and highlights the connection between sociotechnical factors and their agentic and structural contexts, facilitating an understanding of why and when technologies fail and limit.
- Taher R, Hsu CW, Hampshire C, Fialho C, Heaysman C, Stahl D, et al. The safety of digital mental health interventions: Systematic review and recommendations. JMIR Ment Health. 2023;10:e47433. 10.2196/47433.
  - This study reviews the literature to assess how digital mental health interventions (DMHI) assess safety, what risks are reported, and how they are mitigated in both the research and postmarket phases and building on existing recommendations for assessing, reporting, and mitigating safety in the DMHI and standardizing practice.
- Jutai JW, Hatoum F, Bhardwaj D, Hosseini M. Implementation of digital health technologies for older adults: A scoping review. Front Aging. 2024;5:1349520. 10.3389/fragi.2024.1349520.
  - This review aims to analyze research studies on the effectiveness of digital health technologies for older adults to answer the question, "How well do these studies address factors that affect the implementation of technology?" and found common problems with the conceptualization, design, and methodology in studies of digital technology that have contributed to the slow pace of implementation in home care and long-term care.

## Supplementary Information

Below is the link to the electronic supplementary material.

Supplementary file1 (DOCX 17 KB)

Supplementary file2 (DOCX 65 KB)

Supplementary file3 (DOCX 57 KB)

## Data Availability

The authors confirm that the data supporting the findings of this study are available within the article and its Supplementary Materials.
