# Supplementary material for: Digital Tools to Support Mental Health in Later Life: Scoping Review of Systematic Reviews
Source: Curr Psychiatry Rep. 2026 Jun 17;28(1):39. doi: 10.1007/s11920-026-01689-x (PMC13272217; doi:10.1007/s11920-026-01689-x)
Supplement: Supplementary file 1 — Supplementary file1 (DOCX 17 KB) [file 11920_2026_1689_MOESM1_ESM.docx]

**Supplemental file 1.** Search as applied in MEDLINE^1^

| **#** | **Searches** | **Results** |
| --- | --- | --- |
| 1 | exp Mental Disorders/ | 1487676 |
| 2 | exp mental health/ | 67128 |
| 3 | exp Stress, Psychological/ | 156342 |
| 4 | ((mental or psychologic* or psychiatric) adj3 (disorder* or symptom* or stress* or illness or health)).ti,ab,kw. | 420034 |
| 5 | (loneliness or "social isolation").ti,ab,kw. | 25348 |
| 6 | exp telemedicine/ | 47457 |
| 7 | exp digital health/ | 277 |
| 8 | exp Internet-Based Intervention/ | 1237 |
| 9 | exp mobile applications/ | 12491 |
| 10 | ((telemedicine or telephone* or telehealth or telecare or "digital technolog*" or "digital health" or "internet*" or online or "wearable electronic device*" or "e-mental health" or "mobile health" or sensor* or "digital mental health" or smartphone or "mobile health app*" or "social media") adj3 (intervention* or support* or program* or therap* or innovation* or service* or tool*)).ti,ab,kw. | 52913 |
| 11 | exp evaluation study/ | 262154 |
| 12 | exp validation study/ | 109611 |
| 13 | (evaluation or "validation study" or "qualitative evaluation" or efficacy or "randomi?ed control* trial" or "pilot study" or "co-design*" or "co-creat*" or participatory or "mixed-method*" or feasibility or acceptability or "design case study" or "interactive group study" or perspective* or perception* or analys*).ti,ab,kw. | 9227463 |
| 14 | exp aged/ | 3499038 |
| 15 | ((old* or elder* or geriatri* or aged or senior*) adj2 (adult* or person* or people or population* or patient* or subject* or m?n or wom?n)).ti,ab,kw. | 1157646 |
| 16 | 1 or 2 or 3 or 4 or 5 | 1853411 |
| 17 | 6 or 7 or 8 or 9 or 10 | 101148 |
| 18 | 11 or 12 or 13 | 9393642 |
| 19 | 14 or 15 | 4147043 |
| 20 | 16 and 17 and 18 and 19 | 1763 |
| 21 | limit 20 to "all aged (65 and over)" | 1412 |
| 22 | limit 21 to last 15 years | 1286 |

^1^ Ovid MEDLINE(R) Epub Ahead of Print, In Process & Other Non-Indexed Citations, Ovid MEDLINE (R) Daily, and Ovid MEDLINE (R) 1946 - 6 May 2024. Updated on 9 April 2026 to include the last 2 years.
