# Supplementary material for: Digital Tools to Support Mental Health in Later Life: Scoping Review of Systematic Reviews
Source: Curr Psychiatry Rep. 2026 Jun 17;28(1):39. doi: 10.1007/s11920-026-01689-x (PMC13272217; doi:10.1007/s11920-026-01689-x)
Supplement: Supplementary file 2 — Supplementary file2 (DOCX 65 KB) [file 11920_2026_1689_MOESM2_ESM.docx]

**Supplemental file 2.** Quality assessment using modified CASP tool

| Author | Section A: Are the results of the review valid? | | | | | | | | | | Section B: What are the results? | | | |
| --- | --- | --- | --- | --- | --- | --- | --- | --- | --- | --- | --- | --- | --- | --- |
|  | Clearly focused research question | Comments | Right type of papers | Comments | All relevant studies included | Comments | Sufficient quality assessment | Comments | Reasonable to combine results, if done | Comments | Overall results of review | How precise are results? | General comments |  |
| Chae & Lee | Yes | Aims are made clear: ‘…the purpose of our study was to analyze not only cognitive function outcomes, including attention, language, and memory, but also psychosocial outcomes in community‐dwelling patients with MCI or mild dementia after an ICT‐based intervention.’ | Yes | Inclusion of randomized trials fits aim to explore cognitive and psychosocial outcomes | Yes | 4 relevant databases searched; screened list of references from recent reviews; manual search; included studies in Korean and English; no mention of expert consultation | Yes | ‘Two authors independently assessed the quality of the selected studies using the Cochrane risk‐of‐bias tool for randomized  controlled trials.’  Authors note that high risk of bias was considered in interpretation of findings | Yes | Sub-group meta-analyses performed where studies had common outcomes or methods (e.g. studies that administered the Mini-Mental State Examination; interventions lasting >30min per session). Results of individual studies summarised in a detailed table | ‘ICT‐based cognitive interventions significantly improved the Mini‐Mental State  Examination score in the intervention group compared with that in the control  group. Additionally, cognitive training using ICT was significantly effective for a period of more than 30 min, more than 6 weeks, and multi‐domain content. Moreover, a significant reduction in depression was found in the intervention group  compared with that in the control group.’ | Authors note high risk of bias in studies and small sample size of some studies. Heterogeneity of study interventions and measures means that small meta-analyses may be biased | Indeed, of the 44 studies included, for some outcomes of interest (i.e. depression) there are only 5 (11%) eligible for meta-analysis. Authors acknowledge this limitation but readers should still interpret such findings with caution. Review was otherwise well conducted. |  |
| Chastin et al. | Yes | Aims are made clear: ‘**Primary:**  - To assess the effect on total sedentary time and the pattern of accumulation of sedentary time of interventions aimed at  modifying sedentary behaviour in older adults who are 60 years and over compared to control conditions involving either  no intervention or interventions that do not target sedentary behaviour.  **Secondary:**  - To summarise the effects of interventions to reduce sedentary behaviour on quality of life, depression, and health status in  older adults.  - To summarise any evidence on the cost-effectiveness and  unintended consequences of interventions that reduce  sedentary behaviour in older adults.’ | Yes | Inclusion of only RCTs and cluster-RCTs fits with research aims | Yes | 9 relevant databases searched; screened the reference lists of included articles and  contacted authors to identify additional studies; contacted subject matter experts to identify any additional unpublished work; no language restrictions | Yes | Bias assessment done using Cochrane risk of bias tool and results tabulated using GRADE methodology | Yes | included studies were tabulated and grouped by study design, population, outcomes, and setting; summarised in narrative synthesis  Meta-analysis undertaken for outcomes with sufficient data (excluded depression, health status, and quality of life)  Results of individual studies summarised in a detailed table | ‘It is not clear whether interventions to reduce sedentary behaviour are effective at reducing sedentary time in community-dwelling older adults. We are uncertain if these interventions have any impact on the physical or mental health of community-dwelling older adults. There were few studies, and the certainty of the evidence is very low to low, mainly due to inconsistency in findings and imprecision.’ | Authors also note homogeneity of study samples and lack of data to address objectives for the review | The review was well conducted but appears the primary studies included did not facilitate ‘a robust  answer to the research questions of this review’ |  |
| Christensen et al. | Yes | Aims are made clear: ‘…the aim of this review was to conduct a systematic review of the existing research literature, focusing on patients' and providers' experiences of VCs used in the treatment of patients 60+ years with unipolar  depression.’ | Yes | Quantitative, qualitative or mixed methods designs were all included ‘to capture the nuanced picture of experiences’ seems a sensible approach and acknowledges the importance of different types of data  However, the breadth of types of studies, all with their own quality issues, seems to have had its challenges for analysis | Yes | 8 relevant databases searched; reference lists of included articles were searched; included articles written in 5 languages | Yes | Two authors conducted appraisal using CASP; decision to conduct meta-analysis was determined by results of this appraisal | Yes | Meta-analysis was not conducted due to low quality assessment results. Qualitative and summarised in thematic analysis and quantitative data summarised in narrative synthesis, though it’s unclear if a specific method was followed for quantitative  Results of individual studies summarised in a detailed table | ‘Most studies reported positive findings in regard to patients' and providers' experiences when using VCs’ and these were comparable to face-to-face interactions | Low quality appraisal results and other limits to validity and generalisability are discussed. | Given that the review did not aim to assess clinical outcomes, the challenges with methodologies discussed by the authors should perhaps not be surprising. Methods for synthesising data not reported which reduces overall quality of the review. |  |
| Cremers et al. | Yes | Aims are made clear: ‘The aim of this review is to investigate the effectiveness  of a broad range of low-intensity, psychological  interventions on the mental health of older adults through systematic evaluation of the extant  literature and to suggest clinical implications for  the findings.’ | Yes | Broad criteria used to examine wide range of studies for qualitative synthesis: ‘studies considered for inclusion had to measure pre- and post-intervention mental health.’ | Yes | Searches run in databases within 3 host platforms (Ovid, EBSCOhost, and ProQuest). Reference lists of included studies were checked.  Grey literature sought but no information about how. No information on language(s) of articles included. | Yes | MINORS scale used along with 1 item about ethical considerations from CASP. Results of this are discussed and its implications for interpreting the findings are considered in the Discussion | Yes | Studies grouped by type of intervention (e.g. bibliotherapy, iCBT) and relevant information summarised across several tables | ‘Low-intensity psychological interventions can be effective for older adults with mild-to-  moderate mental health problems, but generalizability is constrained.’ | Meta-analysis was not possible due to wide range of outcome measures and inconsistent use of control groups. Generalisability is limited due to diversity of interventions. | Given the broad criteria, this study managed the findings well. Future work may learn from this and provide a more focused review (e.g. focus on few types of interventions or outcome measures) |  |
| de Oliveira et al. | Yes | Aims are made clear: ‘the present study aimed to evaluate the efficacy of interventions by telemedicine on reducing depressive and anxious  symptoms or disorders in older people through a systematic review and meta‐analysis. Moreover, we aimed to compare the efficacy between telemedicine interventions and usual care or waitlist, and among different types of telemedicine on these symptoms.’ | Yes | RCTs, quasi-experimental studies, interrupted time series analysis, pre/post test experiments. These fit with the aim to compare efficacy between telemedicine and usual care/wait list | Yes | Searches run in 7 relevant databases; no language restrictions; reference lists of eligible studies checked for additional articles; grey literature was not sought | Yes | Version 2, Cochrane Collaboration's Risk of Bias Tool for RCTs. Only 19.3% of included studies deemed low risk. Authors discuss insufficiency of evidence to conclude if telemedicine interventions are clinically meaningful | Yes | Only 4 studies selected for meta-analysis due to heterogeneity of sample, 3 of which were judged with ‘some concern’  Relevant information about the 30 individual studies is adequately summarised across several tables | 30 studies demonstrated that telemedicine interventions are feasible for the reduction of depressive and anxious symptoms in older people. However, difficult to draw conclusions about meaningful clinical changes given the heterogeneity of studies | Use of psychotropic drugs not reported in half the studies. This is a potential confounding factor that makes it difficult to understand efficacy of telemedicine interventions, in addition to other risk of bias concerns noted by the authors | Again, the review was well conducted and authors aptly were cautious in drawing conclusions given the heterogeneity of studies and small number included in meta-analysis |  |
| Dworschak et al. | Yes | Aims are made clear: ‘we conducted a systematic review and meta-analysis  on the existing literature on internet-based psychological interventions  for the treatment of common mental disorder symptoms and  psychosocial problems in older adults. We were mainly interested in a)  what kind of interventions already exist and b) how effective they are in  improving older adults' mental health.’ | Yes | RCTs and non-RCTs involving any form of control condition were included. Must have quantitative post-intervention measures of common mental disorder symptoms or psychosocial problems  Controlled pilot studies were included if no results of larger  trials had been published  These criteria seem sensible but may make it difficult to perform a meta-analysis if there is a heterogenous study sample | Yes | Searches run in 4 relevant databases; searches were redone to include sleep disorders and mobile-based interventions.  Articles in 4 languages were included (English, French, German, Italian) which may skew results toward high-income countries but is better than English alone  No mention of searching reference lists of included articles, manual searching, or expert consultation | Can’t tell | Version 2, Cochrane Collaboration's Risk of Bias Tool for RCTs and Cochrane Risk of Bias in Non-Randomized Studies of Interventions Tool  Authors note that blinding of participants and personnel difficult to achieve in psychology research and these domains may yield high risk of bias. 90% of randomized studies had high risk of bias but noted that high risk in 1 domain leads to this result. Would another method of quality assessment have been more appropriate? | Yes | 11 studies included, adequate individual study details summarised in tables.  10 RCTs and 1 non-RCT included. An overall effect size was calculated for all psychological and psychosocial outcomes combined. Pooled post-intervention effects also calculated for depression and anxiety symptoms. Subgroup analysis only performable on overall symptom severity | To answer first research question (what kinds of interventions exist): ‘there exist 11 interventions aiming at improving older adults' common mental disorder symptoms (depression,  anxiety, PTSD) and subclinical psychosocial problems (stress).’  To answer second research question (how effective are they): ‘we found  promising results. Significant large effects of internet-based interventions  for older adults were found for overall symptom severity  (depression, anxiety, PTSD, stress) as well as for depression symptom severity.’ | Research in this field still at early stage. All studies were conducted in Western countries, lack of information about long-term effects. Unable to test for publication bias due to limited # and high heterogeneity of studies | Not sure the choice in quality assessment tools were sensible if the authors questioned their outcome due to nature of psychological research. Makes it difficult to understand the true weight of assessment results and how this impacts the way we interpret findings |  |
| Fernandes et al. | Yes | Aims are made clear: ‘This systematic review aimed to synthesize current evidence on telehealth interventions for adults aged 65 years and older, focusing on their effects on health outcomes, quality of life, and well-being.’ | Yes | Experimental studies, observational studies, qualitative studies, mixed-methods studies, intervention studies, implementation science studies, case studies, and systematic reviews/meta-analyses or scoping reviews that include data related to the impact of telehealth solutions. | Yes | Searches run in 3 databases; included articles written in English, Portuguese or Spanish  No mention of searching grey literature or expert consultation. Searching only 3 databases is on low end of adequate but hopefully inclusion of multiple-language articles makes up for this. | Can’t tell | The methodological quality of the included studies was assessed using the Joanna Briggs Institute (JBI) Critical Appraisal Tools, with specific validated checklists applied according to each study design.  Articles were not excluded based on the JBI score, as the objective was only to examine the rigor of the included studies. It is unclear how the quality assessment impacted the interpretation of results. The results of the assessment are only mentioned once in the Discussion: ‘Furthermore, the moderate methodological quality and the significant heterogeneity of included studies complicate comparisons and affect interpretation of results, limiting generalizability.’ | No | The results are synthesised in a way that doesn’t provide a lot of insight. Effect sizes/significance levels are not provided, and the results are reported at a high level. This is possibly because the inclusion criteria are too broad to allow for meaningful insights about what is effective, for which conditions, and for which outcomes. | ‘Reported benefits included improved physical function, better chronic disease control, greater health knowledge, and reductions in avoidable hospitalizations. Video-based programs showed greater effectiveness, while telephone-only interventions were most useful when combined with remote monitoring. Adherence was strengthened by professional guidance, caregiver support, and real-time feedback.’  There is so much heterogeneity in the included studies that it seems difficult to make meaningful comparisons. | The authors acknowledge the lack of precision: ‘significant heterogeneity of included studies complicate comparisons and affect interpretation of results, limiting generalizability.’ | The review did not report non-significant outcomes but did not mention this in the methods. The authors acknowledge this may introduce bias and I agree. |  |
| Goodarzi et al. | Yes | Aims are made clear: ‘we conducted a systematic review of RCTs reporting the efficacy of virtual interventions for reducing symptoms of depression in older adults.’ | Yes | RCTs comparing the efficacy of any  virtual nonpharmacologic intervention to usual care or any other virtual nonpharmacologic intervention for reducing symptoms of depression in community-dwelling  older adults  depressive symptoms or disorders at baseline or where depression was measured as an outcome’  Meta-analysis wasn’t performed. | Yes | Search developed with experienced librarian and a second librarian conducted a peer review of electronic search strategies. 5 relevant databases searched; 76 grey literature sources sought. No language restrictions; no mention of checking reference lists of included studies or manual searching | Yes | Cochrane Risk of Bias Tool for RCTs was used. ‘Allocation concealment (n = 10 unclear) and blinding  procedures (n = 11 unclear; n = 4 high risk) were  the most important sources of bias’ | Yes | Clinical heterogeneity of studies meant meta-analysis was not possible despite 15 RCTs being included. Adequate individual study details summarised in tables | Review found that virtual interventions are feasible and sometimes demonstrated efficacy. ‘Harm was not reported in any study. However, there was insufficient  evidence to establish whether these interventions lead to clinically meaningful outcomes or durable results for patients. Virtual interventions were delivered via telephone or internet and had varied degrees of involvement from clinicians.’ | Heterogeneity of studies makes it difficult to draw strong conclusions, which the authors acknowledge. Risk of bias and generalisability of study outcomes also acknowledged. | The included studies provide insight but not a deep understanding of the efficacy of virtual care for depression in older adults. Review well conducted overall |  |
| Jin et al. | Yes | Aims are made clear: ‘the present study aimed to  conduct a systematic review of existing studies that examined the effectiveness of technology-based interventions for reducing  loneliness in older adults.’ | Yes | RCTs assessing outcomes of loneliness in a rated scale  RCTs that had no significant effect on loneliness reduction were also included  Meta-analysis performed | Yes | Studies in English and Chinese were included; 5 relevant databases searched in addition to 2 Chinese language databases; reference lists of included articles were checked.  No mention of expert consultation or grey literature sources | Yes | Assessed risk of bias with Cochrane Collaboration’s Tool for Assessment of Risk of Bias. Results clearly reported in results. Few studies displayed low risk of bias; most unclear or high risk | Yes | 6 RCTs included and eligible for meta-analysis. Adequate individual study details summarised in tables  Meta-analysis of the effect of technology-based interventions vs. control groups on loneliness scores among older adults- calculated using random effects model. Subgroup analysis for different intervention types  ‘Subgroup analysis for age, living conditions, follow-up time, and measurement tools were planned; however, there  were not enough trials or information to perform meaningful analyses.’ | Technology-based interventions showed little or no effect on loneliness reduction in older adults  Wide variation in sample size, participant demographics, measures, intervention methods, and duration  Subgroup analysis based on intervention type also showed very uncertain effectiveness | 3 of 6 studies rated low quality, the other 3 rated as moderate quality. Paucity of data from RCTs makes it difficult to draw conclusions from this and other similar works. Authors note that studies did not necessarily target types of older adults most at risk of loneliness | Review appears to be well conducted but authors acknowledge that focus on RCTs only means little evidence to draw from. |  |
| Li et al. | Yes | Aims are made clear: ‘This systematic review and meta-analysis of RCTs and non-randomized studies of interventions (NRSIs) aims to: 1) systematically synthesize the details of all available digital health interventions of older adults with CF, 2) preliminarily evaluate the overall efficacy of these interventions, and 3) evaluate implementation outcomes linked with the delivery of digital health interventions in this population.’ | Yes | Interventional studies comparing digital health interventions with conventional interventions not using digital technologies | Yes | Emphasis on Chinese sources of evidence, but still includes international databases | Yes | ‘For RCTs the risk of bias was assessed by two independent reviewers using the Cochrane collaboration's Risk Interventions Risk of Bias Tool (ROB2)  For NRSIs, assessed the risk of bias using the Cochrane Risk of Bias Assessment Tool: Risk of Bias in Non-randomized Studies of Interventions (ROBINS-I)’  ‘It is critical to emphasize that a substantial proportion (46 %, 6/13) of the included studies exhibited elevated risk of bias, necessitating cautious interpretation of the findings.’ | Can’t tell | ‘Whenever two or more studies reported the same outcome, the meta-analysis was carried out. If the studies were unsuitable for meta-analysis, their outcomes were summarized through narrative syntheses.’  Not sure what the authors mean by ‘same outcome’ as in the same assessment tool or just the same domain e.g. depression | Intervention had a significant effectiveness on improving depressive symptom  Meta-analysis for loneliness not performed due to insufficient intervention data | ‘Many had small sample sizes (77 %, 10/13), which may have limited statistical power and resulted in imprecise effect estimates. Also, the small number of included studies and participants inherently limited the generalizability of our findings, and the lack of long-term follow-up data restricted our ability to assess the sustainability of intervention effects over time. Additionally, some studies introduced methodological concerns that might have increased the risk of bias, due to insufficient details on randomization procedures, blinding, and outcome measures.’ | Mental health was assessed in relatively few studies and often depressive symptoms were used as proxy for mental health when in reality the only other measure (loneliness) was not included in the meta-analysis. |  |
| Morris et al. | Yes | Aims are made clear: ‘The aim of this study was to undertake a systematic literature review of studies that assessed the effectiveness of smart technologies in improving or maintaining the social connectedness of older people living at home.’ | Can’t tell | Given social connectedness is a multidimensional concept, inclusion of some form of intervention study and assessing 1 of 9 pre-defined concepts of social connectedness makes sense. However, synthesising these data may be tricky and authors acknowledge that studies may be missed when being selected based on this.  Defining older people as 45+ years is debatable, when the authors say themselves that MeSH term for ‘middle-aged’ is 45-64 years. The study is meant to be about ‘older people’ so unclear why the authors included ages in the ‘middle-aged’ MeSH term.  Definition of ‘smart technologies’ not clearly described until the results in which the types of interventions included are described. | Can’t tell | 6 relevant databases searched but no other indication that an effort was made to find all relevant studies: English-language only, no mention of: expert consultation, checking included studies’ reference lists, or searching for grey literature | Yes | ‘Downs and Black checklist was utilised to perform  the quality assessment. This checklist has been designed to help determine the quality of randomised and nonrandomised  studies and therefore was appropriate for the aims of the current review.’  Median score of the 18 included studies was 18.5 our of possible 27. Authors cite lack of information about study population, large attrition rates, and lack of blinding where applicable | Yes | Adequate individual study details summarised in tables  Data and study outcomes appear to be summarised in narrative synthesis, though the analysis method was never explicitly stated, outside of the abstract which states ‘a systematic review and critical evaluation of research articles’ | Authors conclude there is emerging evidence to support the use of technology to improve some dimensions of social connectedness as well as other related factors | Authors note that generalisability is limited due to studies’ procedures and lack of sufficient detail about participants and programs  Large number of outcome measures limited comparison between studies | Lack of explicitly stated analysis process, mixed methods of included studies, and questionable rationale for age criteria means results should be interpreted with caution. |  |
| Oh et al. | Yes | Aims are made clear: ‘The overall purpose of this review is to explore how CAI may support older adults, with a focus on its impacts and challenges to gain a deeper understanding of its potential.’  ‘…aimed to (1) review the evidence of the impact of CAI technology on psychosocial and health outcomes related to social connectedness among older adults, and (2) explore the perceived challenges in usability and usefulness associated with CAI technology for older adults, to inform future research and practical decision-making regarding its use for enhancing the quality of life of older adults’ (Figure 1). | Can’t tell | ‘Qualitative, intervention, study protocols, feasibility, design, and participatory design articles were included as well. Eligible studies included those that examined interventions or discussions related to CAI, chatbots, or conversational agents among adults aged 50 and above, with primary outcomes related to social connectedness.’  Not sure what ‘discussions related to CAI’ means – not sure how useful it is to include protocols | Yes | 6 databases were searched. Primary sources or research reports written in English and published in peer-reviewed journals between 2014 and 2024 were included.  Citation searching of included articles was also performed | Can’t tell | ‘Quality appraisal and risk of bias were assessed independently by two authors using the Joanna Briggs Critical Appraisal Tools, evaluating methodological rigor and bias based on study design and implementation’  It is unclear how the quality assessment impacted the interpretation of results. | Yes | Adequate individual study details summarised in tables but a lot of heterogeneity  ‘Our synthesis adopted an exploratory and descriptive approach, comparing and contrasting findings to uncover patterns and variations across studies. We considered both homogeneity and heterogeneity, recognizing differences due to methodological variations, which led to a more comprehensive interpretation. Qualitative data from four mixed-methods studies and one qualitative study were integrated with primarily quantitative results, enhancing interpretation and context. Qualitative insights were embedded within the discussion of key quantitative findings to explain specific aspects of the data.’ | The data and results are presented nicely, but given the breadth of outcomes and points of interest, it means that with only 18 included studies, relevant information may come from only a small number of studies depending on the primary studies’ outcomes of interest  Still not sure how protocols/proposals would provide useful information unless they are reporting on previous findings (e.g. pilot data) as well  Table 4 is difficult to interpret – not sure if all studies covered these domains or if the percentages listed are just from those endorsing the domain? | While social connectedness is the main point of interest, a breadth of outcomes were described at a high level. It’s difficult to know which CAI interventions had the greatest impact but there is valuable information about usability, challenges of CAI in older adults | Some of the conclusions are not really backed by evidence since the design studies didn’t seem to report intervention outcomes – e.g. ‘Additionally, through the inclusion of design studies, it has become clear that involving stakeholders (older adults) in the design of an AI intervention aimed at them will result in a more successful AI intervention.’ |  |
| Posadzki et al. | Yes | Aims are made clear: ‘To assess the effects of ATCS for preventing disease and managing long-term conditions on behavioural change, clinical, process, cognitive, patient-centred and adverse outcomes.  Specific secondary objectives include:  1. determining which type of ATCS is most effective for preventive healthcare and management of long-term conditions;  2. exploring which components of the interventional design  contribute to positive consumer behavioural change;  3. exploring the behaviour change techniques and theoretical models underpinning the ATCS interventions.’ | Yes | ‘Randomised, cluster- and quasi-randomised trials, interrupted time series and controlled before-and-after studies comparing ATCS  interventions, with any control or another ATCS type’  Broad range of study types and outcomes was chosen as authors say it’s the first to review all relevant evidence on broadly defined ATCS interventions; future updates may need a narrower focus. This approach is sensible | Yes | 10 relevant databases searched; grey literature (3 databases); contacted experts in the field; searched reference lists of included studies and relevant previous reviews; searched online trial registers; no language restrictions | Yes | Reported risk of bias in accordance with Cochrane Handbook for  Systematic Reviews of Interventions and Cochrane Consumers  And Communication guidelines. Also reported quality of evidence using GRADE criteria  Eligible studies were included regardless of outcome in risk of bias assessment  Results of assessment clearly reported. Certainty of evidence for most outcomes was low. Downgrading of evidence often due to risk of bias | Yes | Adequate individual study details summarised in tables  Meta-analysis performed when studies were sufficiently similar to provide clinically meaningful result. Otherwise, data were synthesised by grouping by type of intervention or long-term condition, and intervention | 132 trials included across preventive healthcare areas and management of long-term conditions  Effects varied by condition and ATCS type. Only 4 trials reported adverse events  Authors conclude that ‘ATCS interventions can change patients' health behaviours, improve clinical outcomes and increase healthcare uptake with positive effects in several important areas including immunisation, screening, appointment attendance, and adherence to medications or tests. The decision to integrate ATCS interventions in routine healthcare delivery should reflect variations in the certainty of the evidence available  and the size of effects across different conditions, together with the varied nature of ATCS interventions assessed.’ | Authors note lack of adequate detail in studies to replicate findings. Only 2 studies conducted in low-income contexts. Very broad inclusion criteria means that data could be organised in multiple ways. Authors note that high potential for bias in many studies reduces certainty | This review was rigorously conducted but very broad in scope. Results are grouped sensibly but could be analysed in many different ways with potentially different outcomes |  |
| Poscia et al. | Yes | Aims are made clear: ‘our systematic review aims to summarize and update the current knowledge on the effectiveness of the existing interventions for alleviating loneliness and social isolation among older  persons.’ | Yes | Studies published in 5 years since the last review on the topic  Studies measuring effects of interventions targeting social isolation or loneliness in adults aged 65+ years or older adults explicitly targeted and mentioned in study title/abstract  Qualitative or quantitative data included | Yes | Studies in English or Italian; searched 5 relevant databases for articles not included in the most recent review (which should not be, since they only included articles published after the last similar review)  Hand-searched references lists of included articles, but no mention of expert consultation, grey literature | Yes | Used the EPHPP tool for quantitative studies ‘due to its suitability with various study designs’  Qualitative studies evaluated using ‘criteria proposed by Salmon et al. [2013] (theoretical framework, value of study, data  collection, participant description, data analysis, data interpretations)’  Authors stated that majority of quantitative studies had weak ratings and remaining 2 rated moderate. Similar results for qualitative studies. However, issues around blinding of participants may be due to nature of the intervention and thus impact rating. | Yes | Adequate individual study details summarised in tables, grouped by qualitative or quantitative design. Meta-analysis not performed and not justified – presume due to heterogeneity of included studies.  More emphasis on quantitative designs in the narrative, which is sensible given larger number of studies of that type (15 quantitative vs 5 qualitative studies included). Narrative synthesis examined types of interventions  Number of interventions =18 but number of studies included in quantitative findings =15, explained by ‘the fact that Barlett et al. (Bartlett et al., 2013) reported three different demonstration pilot projects in their study and to the fact that Jones et al. (Jones et al., 2015) focused on two different delivery modes of the same intervention (one-to-one and small group).’ | Authors conclude that ‘The results suggested that six out of 11 group interventions (55%), one out of four mixed interventions (25%) and three out of three individual interventions (100%) reported at least one significant finding on social isolation or loneliness. These figures are somewhat diverse to the results of previous reviews published in this field.’ | Authors only updating since last review published 5 years ago. Varied interventions and methods acknowledged as making direct comparisons difficult  Authors acknowledge that study samples were recruited via convenience sampling and thus unlikely to be representative. Homogeneity of study samples is not discussed. | Review did not reach solid conclusions. Authors note that studies in this review did not include all beneficial traits of successful interventions in previous reviews – an important finding and implication for future work. |  |
| Pu et al. | Yes | Aims clearly stated: ‘a systematic review and meta-analysis, based on currently available evidence of RCTs, is needed to clarify the benefits of social robots for older adults… This review focuses on the existing RCTs using social robots in health care of older adults including those with and without cognitive impairment.’ | Yes | RCTs using social robots without restriction of robot type of intervention frequency  Excluded reviews, nonrandomized studies, study protocols, case studies, observational studies, cross-sectional studies, qualitative studies, or pre–post studies without a control group, and conference abstracts without full-text | Yes | 9 databases searched. Searches re-run 10 months after initially done.  Reference lists of included studies were checked and only articles in English were included. No mention of expert consultation or grey literature searches | Yes | Risk of bias assessed with Cochrane Collaboration tool for assessing risk of bias in randomized trials. Results of this were reported in narrative and in a figure. 2 studies were excluded from meta-analysis due to insufficient information and no responses from study authors at request | Yes | Adequate individual study details summarised in tables  Study characteristics described in narrative and then meta-analysis and results reported based on outcome scores of common measures / raw data used across studies  Outcomes not suitable for meta-analysis were described in a narrative review | Authors conclude that ‘Pooled results indicate that social robots have the potential to reduce agitation and anxiety, as well as improve quality of life for older adults. The narrative review indicates that interacting with social robots improves engagement and communication, as well as reduces loneliness, stress responses, and medication use in older adults.’  Meta-analysis did not produce statistical significance, whereas narrative review had more positive findings. | Authors discuss risk of bias and quality of included studies being low. Lack of statistically significant findings from meta-analysis makes it difficult to conclude that positive impacts observed in narrative synthesis are robust. | This review appears well conducted but fits with other reviews in observation that primary studies were low in number and often low in quality/higher risk of bias. |  |
| Rai et al. | Yes | Aims are clearly stated: ‘we set out to undertake a systematic review to: 1) describe the current digital technologies to support or prevent social isolation or loneliness in people with dementia, 2) summate evidence surrounding the effectiveness of these interventions, and 3) assess the reported study quality alongside readiness for implementation.’ | Yes | Any trial design and study type included to capture interventions at an early stage of development, as long as a measure of social isolation/ loneliness was assessed and digital technology was an essential component in overall design | Yes | 5 relevant databases searched in consultation with medical librarian  Studies in English or Dutch included  No mention of checking reference lists of included articles, or search of grey literature | Yes | MMAT used – authors note this is a different tool from that proposed in the original review protocol. Allowed for a single tool to be used for all included studies regardless of design  Authors also appraised the ‘readiness for implementation’ based on availability and accessibility of the technologies  Only 20% of studies deemed high quality; 40% medium to high; 40% deemed medium to low quality | Yes | Mostly individual study details summarised in tables, but critically, intervention durations and individual study designs not reported consistently  Results summarised thematically in narrative synthesis for common themes identified across all studies (e.g. quality of life, social connectedness measures)  No meta-analysis | Authors conclude that ‘All technologies demonstrate some level of improvements on measures of [quality of life] with a number of improvements across outcomes regarding social inclusion, social isolation, and loneliness.’ | Authors discuss that it is difficult to directly compare results due to variation in technology maturity, content, and evaluation approaches  Also note limitations of included studies – lack of reported information on theoretical underpinnings, co-design with people with dementia, and complex technologies with many aspects, thus making it difficult to assess which part(s) are effective | This is one of few reviews to examine the readiness for implementation among interventions studied, an important consideration for digital interventions  Lack of information about intervention duration and study designs are key missing elements to understand the rigor of the studies included. |  |
| Riadi et al. | Yes | Aims are clearly reported: ‘This systematic review aims to investigate the evidence supporting the use of digital mental health interventions for treating and preventing symptoms of depression and anxiety in adults older than 50 years. This systematic review aims to gain insight into the various designs and aspects of digital mental health interventions for older adults, and to synthesise methodological findings from randomised controlled trials with older adult participants to extract important information to conduct future randomised controlled trials for this population.’ | Can’t tell | RCTs of ‘of digital mental health interventions for adults older than 50 years for their depressive or anxiety symptoms. The reason for this younger age cut-off for this population is because there are few studies that include the older population when looking at digital or technology-based health interventions. By lowering the age eligibility criteria, we hope to gather as much evidence as possible for the older population.’  Evidence for the older population may not be robust if the intervention was not developed or tested with older adults in mind | Yes | 3 relevant databases searched with no language restrictions. Reference lists of included articles were searched.  No mention of searching grey literature or expert consultation. Searching only 3 databases is on low end of adequate but hopefully inclusion of any-language articles makes up for this. | Yes | Quality assessed using revised Cochrane risk-of-bias tool for randomised trials (version 2)  Nearly 20% of studies at high risk of bias, 41% at some risk. Many studies noted to have high dropout rates, contributing to missing data | Yes | Adequate individual study details summarised in tables  Meta-analysis not deemed appropriate due to high degree of heterogeneity in outcomes and measurement in included studies  Narrative synthesis was conducted instead, organised by type of digital mental health intervention. Characteristics of each type, and their effectiveness in addressing depressive and anxiety symptoms discussed | Authors conclude that: ‘Despite the apparent potential of digital technology supporting mental health, insufficient evidence suggests that this potential is not being fully realised, with uptake being scarce and outcomes being largely anecdotal and unpublished. Thus authors provide critique of existing literature base and recommendations for characteristics of future interventions | Only 17 studies included in review, 2 being pilot studies  No meta-analysis conducted and no studies had qualitative components. Thus this study provides more of a critical analysis / methodological considerations and recommendations than interpretation of the findings. | In line with other reviews, this one found more questions than answers in regard to its aims. Emphasis made on the fact that digital mental health research is still in its infancy – is this true, or are quality of primary studies simply not telling us what we need to know? |  |
| Ronzi et al. | Yes | Aims are clear: ‘What is the empirical evidence on the impact on health and wellbeing of interventions which foster respect and social inclusion in community-residing older adults? The aims were to (i) identify and understand the health impacts of interventions that aim to promote respect and social inclusion among older people and (ii) to elucidate the complex pathways that may lead to improved health outcomes.’ | Yes | ‘All empirical study designs including quantitative designs (randomised and non-randomised controlled studies, before and after studies), mixed methods design and qualitative designs were eligible for the review.’  Case studies only included if key details were mentioned (sampling techniques, data collection methods, results/ analysis of health impacts) | Yes | 8 relevant databases searched; 7 grey literature sources searched; checked reference lists of relevant papers; contacted topic experts  Only English language papers were included | Yes | Global assessment of all studies given in summary tables  Quantitative studies / quantitative elements of mixed method studies were assessed using the LQATs  Qualitative studies / qualitative elements of mixed methods studies were appraised using an adapted version of Harden et al. and Mays and Pope tools. Global assessment of validity was made based on results of this scale  12 studies rated as high and medium-high risk of bias; 12 studies as moderate; and 21 as low or low-moderate risk of bias | Yes | Adequate individual study details summarised in tables  Meta-analysis not possible with broad focus and varied study designs/outcomes  Narrative synthesis done instead, grouped by type of intervention. Harvest plots to represent quantitative findings  Applied pre-developed logic models for 2 types of interventions  Diagrams developed to present descriptive overview of evidence for each intervention type | 40 studies included.  ‘Music and singing, intergenerational interventions, art and culture and multi-activity interventions were associated with an overall positive impact on health outcomes’  Qualitative studies offered insight into mediating factors that may lead to improved outcomes | All studies conducted in high and upper middle-income countries  Most studies (n = 24) deemed high / moderate risk of bias  Authors note that evidence is based on studies with heterogeneous methodologies  Findings therefore need to be interpreted with caution | The results of this study are informative but there is a lot going on, potentially overcomplicating the findings. A lot of visuals and incorporation of the authors’ predefined logic model give the reader a lot to make sense of. |  |
| Shah et al. | Yes | Aims are clearly described: ‘The primary objective of this study is to assess the effectiveness of DTIs in reducing loneliness in adults. The secondary objective is to identify DTIs that are used to reduce loneliness in adults.’  The authors appear to have included any-age adults but resulting studies ultimately made this an older adult-focused review, which explains the discrepancy in the article’s title | Yes | Included interventional studies, randomised and non-randomised  This is presumably to capture more evidence from a small evidence base than if only including RCTs but may make meta-analysis difficult | Can’t tell | 5 relevant databases were searched; search developed with support of a librarian  English language only; no mention of grey literature, expert consultation, or checking reference lists of included studies. Thus unclear if adequate effort was made to find all relevant articles. | Yes | Quality assessed using GRADE criteria and risk of bias with Cochrane guidelines. Some tests of bias were not possible due to small number of studies eligible for meta-analysis  High risk of bias noted in attrition and other forms of bias. Most studies only reported within-group changes and not between-group changes, suggesting weak quality of reporting of results / analysis of these studies | Yes | Adequate individual study details summarised in tables  Narrative synthesis (narrative summary) for all 6 included studies and statistical synthesis (meta-analysis) for 5 studies similar enough to do so (5 clinical trials vs 1 pre-post study). | 6 studies included in analysis.  ‘The narrative summary of 6 studies included in our review showed a reduction in loneliness in the intervention  groups at follow-up compared with baseline (Table 2). However, our meta-analysis of 5 clinical trials with follow-up  measurements at 3, 4, and 6 months showed no statistically  significant pooled effect estimates…’  Authors note that although it was not statistically significant, summary effect size at 4-month follow-up was better than effect size at 3- and 6-month follow-ups | Noted by authors: Quality of evidence was very low to moderate, high heterogeneity between studies. High proportion of female participants and low total number of participants in studies.  Loneliness is influenced by culture, gender, and age, and these factors could have contributed to the pooled estimates being not statistically significant in our  meta-analysis. | Interesting that even though adults aged 18+ years were eligible, this review only found eligible studies that included older adults. Once again, quality of the primary studies is questioned. Authors required a minimum intervention duration of 3 months, which is useful for exclusion of studies not showing long-term outcomes but means that potentially relevant articles may have been excluded in an already small evidence base. |  |
| Silva et al. | Yes | Clearly described aims: ‘Specifically, this systematic review aims to address  this question: What is the effectiveness of technology-mediated dance interventions in improving psychosocial variables in older adults (+65)?’  Psychosocial variables of interest are described as: standard measures to assess quality of life, falls/fear of falling, and life satisfaction (mood, self-efficacy, fatigue) | Yes | RCTs only as this is considered ‘gold standard’ for assessing the evidence of efficacy of interventions | Yes | 6 databases searched, as well as grey literature repositories. Search extended to additional databases. Not all databases are named but those named appear relevant and sensible.  English language only; searched reference lists of included articles and other systematic reviews. No mention of expert consultation. | Yes | Risk of bias was assessed using Cochrane guidelines  Results showed a high risk of bias for majority of studies. Only 1 study deemed moderate quality, the rest of low quality. Authors note their concern over small sample sizes, high attrition rates for nearly all studies | Yes | Adequate individual study details summarised in tables  Study characteristics summarised and common characteristics described.  Meta-analysis conducted where studies had adequate homogeneity to do so. | 6 articles from 5 studies were included.  ‘None of the studies considered  psychosocial factors as primary outcomes. Secondary outcomes assessed fear of falling, depression,  and training enjoyment, but no study showed evidence of an effective impact on these variables. The meta-analysis  revealed low quality evidence that there was little or no difference above that of the comparison  groups for fear of falling … Similarly, there was little or no difference on depression.’ | Authors are clear that precision of results is questionable. Inclusion of only 5 studies with varied psychosocial variables of interest means that evidence base is potentially too small and weak to draw any conclusions. | Authors note that low quality of studies was noted in another similar review |  |
| van den Berg et al. | Yes | Aims are clear but there are many, possibly losing value if focus cannot be maintained: ‘The aim of this systematic literature review is to answer the following research questions: 1. Is it feasible to support healthcare of older patients with chronic diseases in their homes with telemedical systems? 2. For which specific diseases/ disease groups have telemedicine healthcare concepts been developed? 3. What are the respective applications, modalities, and target parameters of telemedicine interventions? 4. Can determinants of successful telemedicine applications be identified? 5. How good is the acceptance of telemedicine among older patients and among their caregivers? Can specific factors be identified which positively or adversely influence acceptance?’ | Yes | Controlled design studies analysing telemedicine interventions involving patients aged 60+ years  Only studies from 2007-2012 (the year review was conducted) to reduce heterogeneity of studies due to technological advancements/access developments | Can’t tell | 3 relevant databases searched; English language only.  No mention of expert consultation, checking reference lists of included studies, or searching grey literature. Given only 3 databases were searched, seems unlikely that adequate effort was made to identify all relevant studies. | No | No mention of quality assessment being undertaken. Again, for systematic reviews expectations in 2012 may have been different. | Can’t tell | Adequate individual study details summarised in tables  Articles summarised in narrative synthesis though the methods for this are not clearly described. No mention of meta-analysis and why or why not it was not conducted. | 68 articles included in the review.  ‘The studies show predominantly positive results with a clear trend towards better results for “behavioral” endpoints, e.g. adherence to medication or diet, and self-efficacy compared to results for medical outcomes (e.g. blood pressure, or mortality), quality of life, and economic outcomes (e.g. costs or hospitalization).’ | Authors note that ‘in 26 of 68 included studies, patients with characteristic limitations for older patients (e.g. cognitive and visual impairment, communication barriers, hearing problems) were excluded.’ This is presumably a comment on the quality of evidence provided in studies but is not clearly explained. | Many included studies in this review and lack of some key elements for high quality systematic reviews (quality assessment, adequate search for relevant literature) mean the findings from this review need to be interpreted with caution. |  |

CASP indicates Critical Appraisal Skills Programme; MCI, mild cognitive impairment; ICT, information and communication technology; RCT, randomised controlled trial; GRADE, Grading of Recommendations, Assessment, Development, and Evaluations; VC, video consultation; MINORS, Methodological Index for Non-Randomized Studies; iCBT, internet cognitive-behavioural therapy; ATCS, automated telephone communication systems; EPHPP, Effective Public Health Practice Project; MMAT, Mixed Methods Appraisal Tool; LQATs, Liverpool Quality Assessment Tools; DTI, digital technology intervention
