# Supplementary material for: Digital Tools to Support Mental Health in Later Life: Scoping Review of Systematic Reviews
Source: Curr Psychiatry Rep. 2026 Jun 17;28(1):39. doi: 10.1007/s11920-026-01689-x (PMC13272217; doi:10.1007/s11920-026-01689-x)
Supplement: Supplementary file 3 — Supplementary file3 (DOCX 57 KB) [file 11920_2026_1689_MOESM3_ESM.docx]

**Supplemental file 3.** Overlapping of primary studies across included systematic reviews

| Relevant primary studies | Systematic reviews included (n=22) | | | | | | | | | | | | | | | | | | | | | |
| --- | --- | --- | --- | --- | --- | --- | --- | --- | --- | --- | --- | --- | --- | --- | --- | --- | --- | --- | --- | --- | --- | --- |
|  | Chae & Lee | Chastin | Christensen | Cremers | De Oliveira | Dworschak | Fernandes | Goodarzi | Jin | Li | Morris | Oh | Posadzki | Poscia | Pu | Rai | Riadi | Ronzi | Shah | Silva | Van den Berg | Total no. times included |
| Galante 2007 | 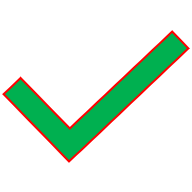 |  |  |  |  |  |  |  |  |  |  |  |  |  |  |  |  |  |  |  |  | 1 |
| Lee 2013 | 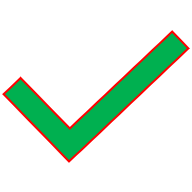 |  |  |  |  |  |  |  |  |  |  |  |  |  |  |  |  |  |  |  |  | 1 |
| Silva 2017 | 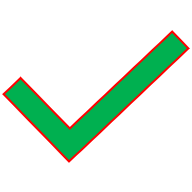 |  |  |  |  |  |  |  |  |  |  |  |  |  |  |  |  |  |  |  |  | 1 |
| Barone 2017 |  | 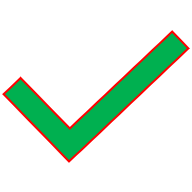 |  |  |  |  |  |  |  |  |  |  |  |  |  |  |  |  |  |  |  | 1 |
| Choi 2014 |  |  | 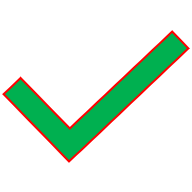 |  |  |  |  |  |  |  |  |  |  |  |  |  |  |  |  |  |  | 1 |
| Jang 2014 |  |  | 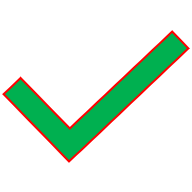 |  |  |  |  |  |  |  |  |  |  |  |  |  |  |  |  |  |  | 1 |
| Arnaert 2007 |  |  | 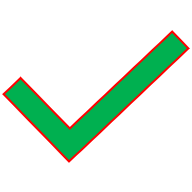 |  |  |  |  |  |  |  |  |  |  |  |  |  |  |  |  |  |  | 1 |
| Jones 2016 |  |  |  | 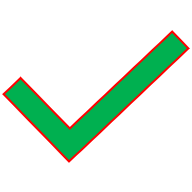 | 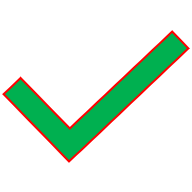 | 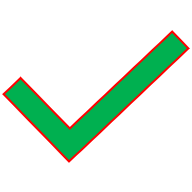 |  | 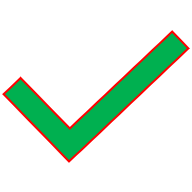 |  |  |  |  |  |  |  |  |  |  |  |  |  | 4 |
| McMurchie 2013 |  |  |  | 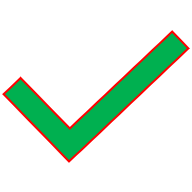 |  |  |  |  |  |  |  |  |  |  |  |  |  |  |  |  |  | 1 |
| Silfvernagel 2018 |  |  |  | 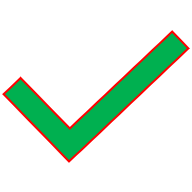 | 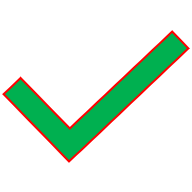 | 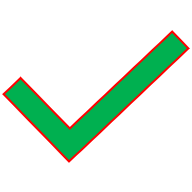 |  | 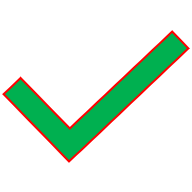 |  |  |  |  |  |  |  |  |  |  |  |  |  | 4 |
| Zou 2012 |  |  |  | 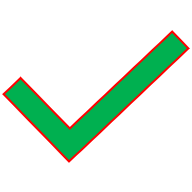 |  |  |  |  |  |  |  |  |  |  |  |  |  |  |  |  |  | 1 |
| Bakas 2018 |  |  |  |  | 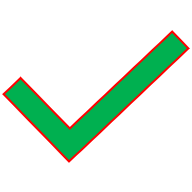 |  |  |  |  |  |  |  |  |  |  |  |  |  |  |  |  | 1 |
| Brenes 2012 |  |  |  |  | 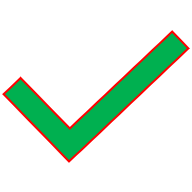 |  |  | 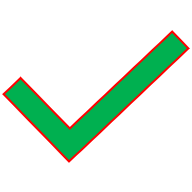 |  |  |  |  |  |  |  |  |  |  |  |  |  | 2 |
| Choi 2020 |  |  |  |  | 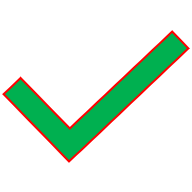 |  |  |  |  |  |  |  |  |  |  |  |  |  |  |  |  | 1 |
| Dear 2015 |  |  |  |  | 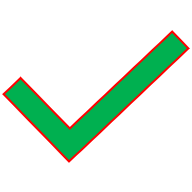 | 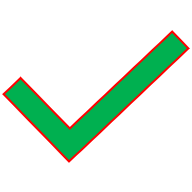 |  | 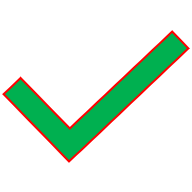 |  |  |  |  |  |  |  |  | 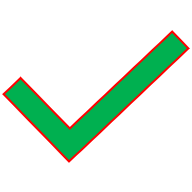 |  |  |  |  | 4 |
| Gellis 2012 |  |  |  |  | 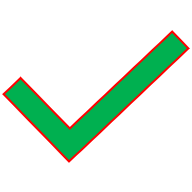 |  |  |  |  |  |  |  |  |  |  |  |  |  |  |  | 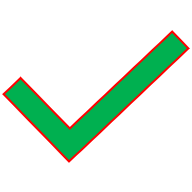 | 2 |
| Gellis 2014 |  |  |  |  | 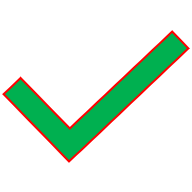 |  |  | 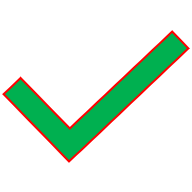 |  |  |  |  |  |  |  |  |  |  |  |  |  | 2 |
| Gould 2019 |  |  |  |  | 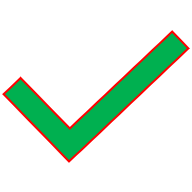 |  |  | 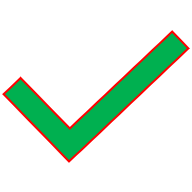 |  |  |  |  |  |  |  |  |  |  |  |  |  | 2 |
| Gustafson 2021 |  |  |  |  | 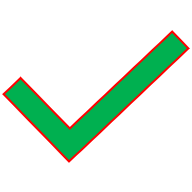 |  |  | 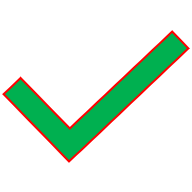 |  |  |  |  |  |  |  |  |  |  |  |  |  | 2 |
| Hartke 2003 |  |  |  |  | 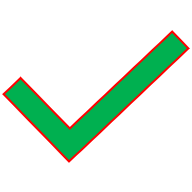 |  |  | 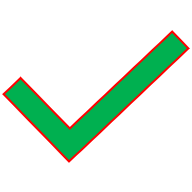 |  |  |  |  |  |  |  |  |  |  |  |  |  | 2 |
| Kenealy 2015 |  |  |  |  | 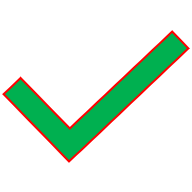 |  |  |  |  |  |  |  |  |  |  |  |  |  |  |  |  | 1 |
| Kornblith 2006 |  |  |  |  | 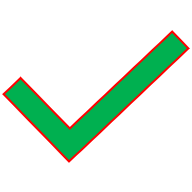 |  |  | 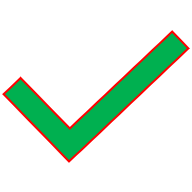 |  |  |  |  |  |  |  |  |  |  |  |  |  | 2 |
| Mavandadi 2015 |  |  |  |  | 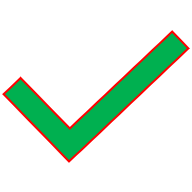 |  |  | 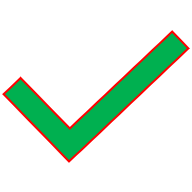 |  |  |  |  |  |  |  |  |  |  |  |  |  | 2 |
| Read 2020 |  |  |  |  | 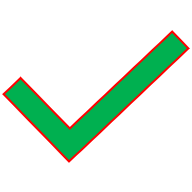 |  |  | 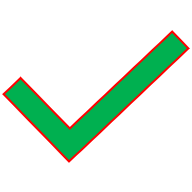 |  |  |  |  |  |  |  |  |  |  |  |  |  | 2 |
| Riegel 2006 |  |  |  |  | 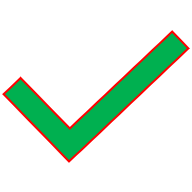 |  |  |  |  |  |  |  |  |  |  |  |  |  |  |  |  | 1 |
| Schoene 2015 |  |  |  |  | 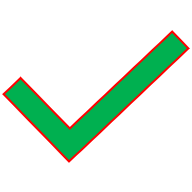 |  |  |  |  |  |  |  |  |  |  |  |  |  |  | 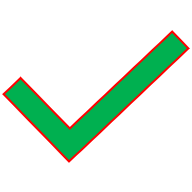 |  | 2 |
| Schwarz 2008 |  |  |  |  | 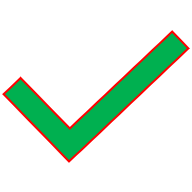 |  |  |  |  |  |  |  |  |  |  |  |  |  |  |  |  | 1 |
| Shapira 2021 |  |  |  |  | 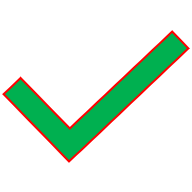 |  |  | 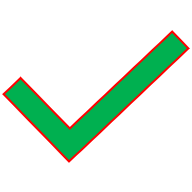 |  |  |  |  |  |  |  |  |  |  |  |  |  | 2 |
| Stahl 2020 |  |  |  |  | 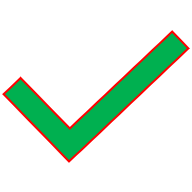 |  |  |  |  |  |  |  |  |  |  |  |  |  |  |  |  | 1 |
| Titov 2016 |  |  |  |  | 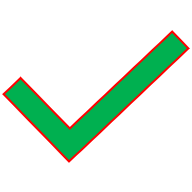 | 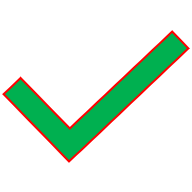 |  | 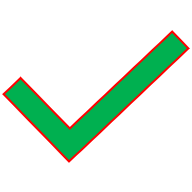 |  |  |  |  |  |  |  |  |  |  |  |  |  | 3 |
| Tomasino 2017 |  |  |  |  | 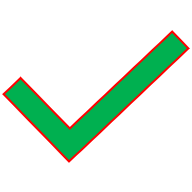 | 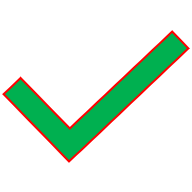 |  |  |  |  |  |  |  |  |  |  |  |  |  |  |  | 2 |
| Villani 2014 |  |  |  |  | 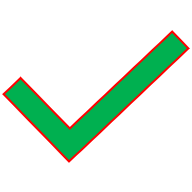 |  |  |  |  |  |  |  |  |  |  |  |  |  |  |  |  | 1 |
| Wong 2021 |  |  |  |  | 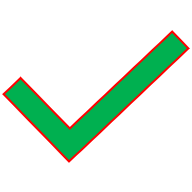 |  |  |  |  |  |  |  |  |  |  |  |  |  |  |  |  | 1 |
| Killen 2015 |  |  |  |  |  | 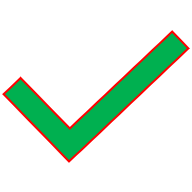 |  |  |  |  |  |  |  |  |  |  |  |  |  |  |  | 1 |
| Knaevelsrud 2017 |  |  |  |  |  | 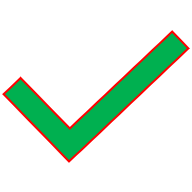 |  |  |  |  |  |  |  |  |  |  | 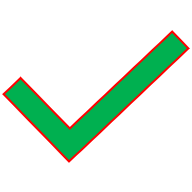 |  |  |  |  | 2 |
| Titov 2015 |  |  |  |  | 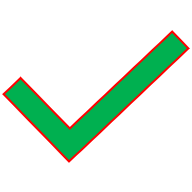 | 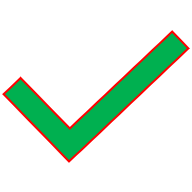 |  | 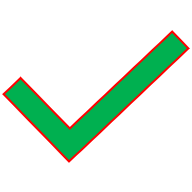 |  |  |  |  |  |  |  |  |  |  |  |  |  | 3 |
| Wahbeh 2018 |  |  |  |  |  | 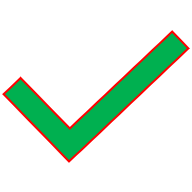 |  |  |  |  |  |  |  |  |  |  |  |  |  |  |  | 1 |
| Brenes 2017 |  |  |  |  |  |  |  | 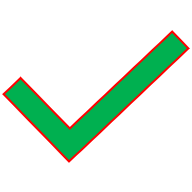 |  |  |  |  |  |  |  |  |  |  |  |  |  | 1 |
| Brenes 2015 |  |  |  |  |  |  |  | 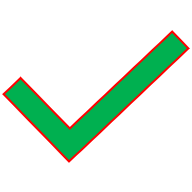 |  |  |  |  |  |  |  |  |  |  |  |  |  | 1 |
| Shapira 2007 |  |  |  |  |  |  |  |  | 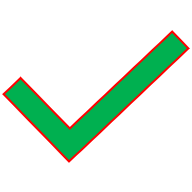 |  |  |  |  |  |  |  |  |  |  |  |  | 1 |
| Hind 2014 |  |  |  |  |  |  |  |  | 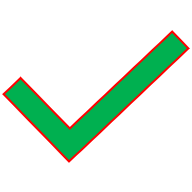 |  |  |  |  | 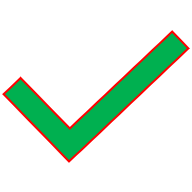 |  |  |  |  |  |  |  | 2 |
| Billip 2001 |  |  |  |  |  |  |  |  |  |  | 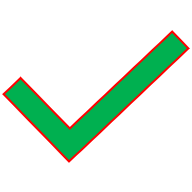 |  |  |  |  |  |  |  |  |  |  | 1 |
| Bond 2010 |  |  |  |  |  |  |  |  |  |  | 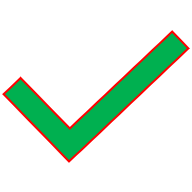 |  |  |  |  |  |  |  |  |  |  | 1 |
| Fokkema 2007 |  |  |  |  |  |  |  |  |  |  | 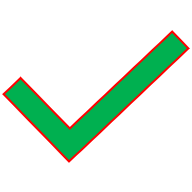 |  |  |  |  |  |  |  |  |  |  | 1 |
| Kahlbaugh 2011 |  |  |  |  |  |  |  |  |  |  | 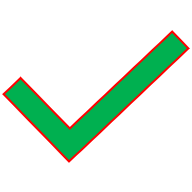 |  |  |  |  |  |  |  |  |  |  | 1 |
| Mahoney 2003 |  |  |  |  |  |  |  |  |  |  | 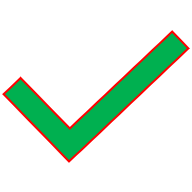 |  |  |  |  |  |  |  |  |  |  | 1 |
| Torp 2008 |  |  |  |  |  |  |  |  |  |  | 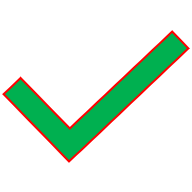 |  |  |  |  |  |  |  |  |  |  | 1 |
| Sparrow 2011 |  |  |  |  |  |  |  |  |  |  |  |  | 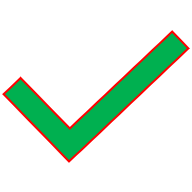 |  |  |  | 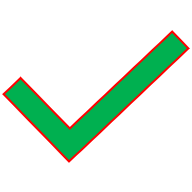 |  |  |  |  | 2 |
| Van Der Heide 2012 |  |  |  |  |  |  |  |  |  |  |  |  |  | 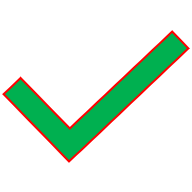 |  |  |  |  | 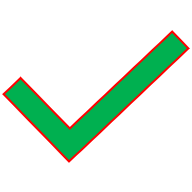 |  |  | 2 |
| Jones 2015 (1) |  |  |  |  |  |  |  |  |  |  |  |  |  | 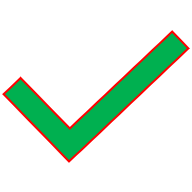 |  |  |  |  |  |  |  | 1 |
| Jones 2015 (2) |  |  |  |  |  |  |  |  |  |  |  |  |  | 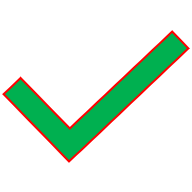 |  |  |  |  |  |  |  | 1 |
| Broadbent 2014 |  |  |  |  |  |  |  |  |  |  |  |  |  |  | 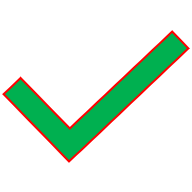 |  |  |  |  |  |  | 1 |
| Tanaka 2012 |  |  |  |  |  |  |  |  |  |  |  |  |  |  | 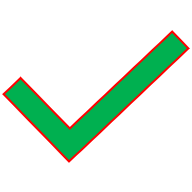 |  |  |  |  |  |  | 1 |
| Demiris 2017 |  |  |  |  |  |  |  |  |  |  |  | 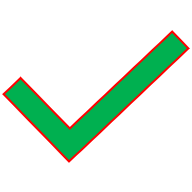 |  |  |  | 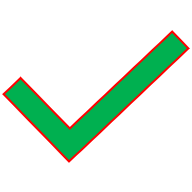 |  |  |  |  |  | 2 |
| Fields 2021 |  |  |  |  |  |  |  |  |  |  |  |  |  |  |  | 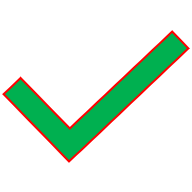 |  |  |  |  |  | 1 |
| Goodman-Casanova 2020 |  |  |  |  |  |  |  |  |  |  |  |  |  |  |  | 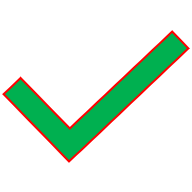 |  |  |  |  |  | 1 |
| Rendon 2012 |  |  |  |  |  |  |  |  |  |  |  |  |  |  |  |  | 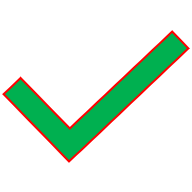 |  |  |  |  | 1 |
| Slegers 2008 |  |  |  |  |  |  |  |  |  |  |  |  |  |  |  |  |  | 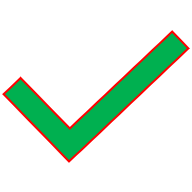 |  |  |  | 1 |
| Woodward 2011 |  |  |  |  |  |  |  |  |  |  |  |  |  |  |  |  |  | 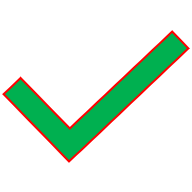 |  |  |  | 1 |
| Woodward 2012 |  |  |  |  |  |  |  |  |  |  |  |  |  |  |  |  |  | 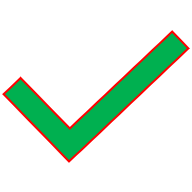 |  |  |  | 1 |
| Schlag 2011 |  |  |  |  |  |  |  |  |  |  |  |  |  |  |  |  |  | 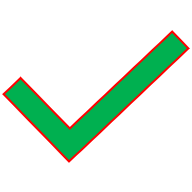 |  |  |  | 1 |
| Larsson 2016 |  |  |  |  |  |  |  |  |  |  |  |  |  |  |  |  |  |  | 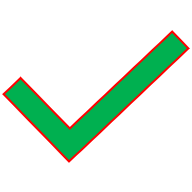 |  |  | 1 |
| Czaja 2018 |  |  |  |  |  |  |  |  |  |  |  |  |  |  |  |  |  |  | 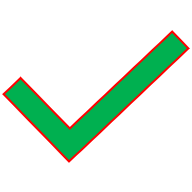 |  |  | 1 |
| Eggenberger 2015 |  |  |  |  |  |  |  |  |  |  |  |  |  |  |  |  |  |  |  | 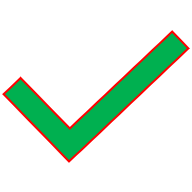 |  | 1 |
| Eggenberger 2016 |  |  |  |  |  |  |  |  |  |  |  |  |  |  |  |  |  |  |  | 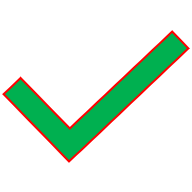 |  | 1 |
| Schoene 2013 |  |  |  |  |  |  |  |  |  |  |  |  |  |  |  |  |  |  |  | 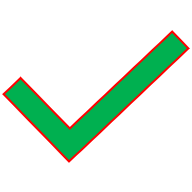 |  | 1 |
| Wong 2024 |  |  |  |  |  |  |  |  |  | 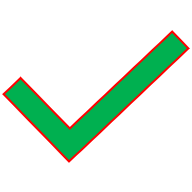 |  |  |  |  |  |  |  |  |  |  |  | 1 |
| Zhu 2023 |  |  |  |  |  |  |  |  |  | 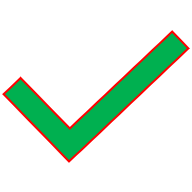 |  |  |  |  |  |  |  |  |  |  |  | 1 |
| Kramer 2022 |  |  |  |  |  |  |  |  |  |  |  | 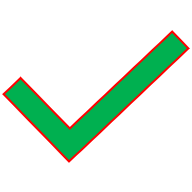 |  |  |  |  |  |  |  |  |  | 1 |
| De Luca 2021 |  |  |  |  |  |  | 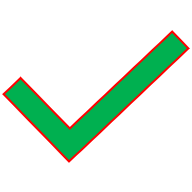 |  |  |  |  |  |  |  |  |  |  |  |  |  |  | 1 |
| Bergerot 2023 |  |  |  |  |  |  |  |  |  |  |  |  |  |  |  |  |  |  |  |  |  | 1 |
| Lang 2021 |  |  |  |  |  |  |  |  |  |  |  |  |  |  |  |  |  |  |  |  |  | 1 |
| Yerlikaya 2021 |  |  |  |  |  |  |  |  |  |  |  |  |  |  |  |  |  |  |  |  |  | 1 |
| Uemura 2024 |  |  |  |  |  |  |  |  |  |  |  |  |  |  |  |  |  |  |  |  |  | 1 |
| Total no. of studies included^1^ | 3 | 1 | 3 | 4 | 24 | 9 | 5 | 16 | 2 | 2 | 6 | 2 | 1 | 4 | 2 | 3 | 4 | 4 | 3 | 4 | 1 |  |

^1^ Primary studies within the reviews that met criteria for inclusion in the current review.
